# Synergistic Enhancement of Rice Yield and Quality by Combined Slow-Release Fertilizer and Urea Under Straw Incorporation Through Optimized Grain Filling and Starch Biosynthesis

**DOI:** 10.3390/plants15172591

**Published:** 2026-08-25

**Authors:** Guan Wang, Bowen Shi, Zixian Jiang, Zichen Liu, Dongchao Wang, Ping Tian, Meiying Yang, Zhihai Wu

**Affiliations:** 1 Faculty of Agronomy, Jilin Agricultural University/Jilin Green and High Quality Japonica Rice Engineering Research Center, Changchun 130118, China; wxkzbwg@163.com (G.W.); 17767979300@163.com (B.S.); 20230789@mails.jlau.edu.cn (Z.J.); l3307185039@163.com (Z.L.); dongchaowang@mails.jlau.edu.cn (D.W.); tianping@jlau.edu.cn (P.T.); 2 College of Life Science, Jilin Agricultural University, Changchun 130118, China; jlaumeiying@163.com

**Keywords:** japonica rice, slow-release fertilizer, combined nitrogen management, straw incorporation, nitrogen use efficiency, starch physicochemical properties, cold agricultural region

## Abstract

Background: In Northeast China’s cold rice (Mollisol) regions, low temperatures slow straw decomposition, causing early microbial nitrogen (N) immobilization that competes with crop demand. Delayed N release from slow-release fertilizer (SRF) exacerbates this deficit, hindering high yield and grain quality. Methods: A two-year (2024–2025) pool planting experiment was conducted on rice ‘Jinongda 667’ with a total N application rate of 150 kg ha^−1^. Six treatments were established: a 7:3 blend of slow-release fertilizer and urea, alongside controls of conventional urea and slow-release fertilizer alone, under both straw removal and incorporation conditions. Results: This blend increased grain yield by 6.64–7.75% over conventional urea, achieving the highest yield among all treatments, whereas SRF alone under straw incorporation reduced yield by 7.57% relative to N + S. The 30% urea (45 kg N ha^−1^) alleviated immobilization deficit (15–30 kg N ha^−1^) during the tillering-to-jointing stage, promoting root growth and panicle formation, while 70% SRF sustained N supply during mid-to-late stages, delaying senescence and enhancing photosynthesis. At peak grain filling, SSS and SBE activities increased by 94.01% and 10.53–11.08%, respectively. Under straw incorporation, it reduced chalky grain rate by 4.56–35.55% and chalkiness by 1.77–41.86%, increased protein content by 0.47–6.95% and gel consistency by 43.59–52.99%, decreased amylose by 2.2–5.77%, and optimized RVA profiles. Conclusions: The 7:3 blend synchronizes N supply with crop demand through temporal complementarity with straw nutrient dynamics, offering a one-time fertilization strategy for achieving high yield and quality under straw incorporation. However, given inter-annual variation (2024–2025) in tillering and N accumulation, its performance may be sensitive to climatic fluctuations, warranting further validation under diverse conditions.

## 1. Introduction

Rice (*Oryza sativa* L.) is the primary staple food for over half of the global population [1]. China is the world’s largest rice-producing country, with both its rice cultivation area and total production ranking among the highest worldwide [2]. Northeast China is the core japonica rice-producing region in China, accounting for more than 50% of the national rice cultivation area and total production, and thus plays a crucial role in ensuring national staple food security [3]. In Northeast China, long, cold winters with temperatures below −20 °C and a low effective accumulated temperature result in slow straw decomposition. Consequently, the nutrient-release patterns of slow-release fertilizers and the decomposition dynamics of straw may differ significantly from those observed in other rice-growing regions [4]. However, most existing studies have focused on the rice-growing regions of the middle and lower reaches of the Yangtze River and plateau rice-growing regions, whereas research on the synergistic effects of combined slow-release fertilizer application and straw incorporation in the cold rice region of Northeast China remains extremely scarce.

Slow-release fertilizer (SRF) is widely used in rice production because its nutrient-release rate is slower than that of conventional fertilizers and better matches crop nutrient demand patterns [5]. Unlike conventional urea, which rapidly releases a large amount of nutrients after soil application and is susceptible to leaching and volatilization losses, slow-release fertilizer regulates nutrient release through the physical barrier of its coating material, thereby significantly reducing nitrogen losses and improving fertilizer use efficiency [6]. Over a two-year period, the controlled-release blended fertilizer (BBF) simultaneously improved yield (10.8–11.0 t ha^−1^) and grain quality. However, a major limitation of the sole application of slow-release fertilizer is its insufficient nutrient release during the early growth stage. The relatively low nitrogen release from slow-release fertilizer during the tillering stage may fail to meet the high N demand of rice for tiller initiation, thereby inhibiting the formation of productive tillers [5]. Recent studies under straw-incorporated conditions have also confirmed that slow-release fertilizer alone cannot fully compensate for the early N deficit caused by microbial immobilization [7]. To address this issue, researchers have begun to explore the strategy of combining slow-release fertilizer with urea, utilizing urea to supply readily available nitrogen in the early stage and slow-release fertilizer to ensure sustained nitrogen supply during the mid-to-late stages. A single basal application of 70% polymer-coated urea plus 30% urea has been shown to maximize seasonal dry matter accumulation, nitrogen uptake, and grain yield in rice, balancing the early nitrogen supply at tillering and the sustained supply in later periods [8]. Recent field studies have further corroborated this finding: a single basal application of urea combined with 40-day and 90-day slow-release urea achieved grain yields 9.6% higher than conventional fertilization, alongside consistently greater effective panicle numbers [9]. Collectively, these studies consistently indicate that the 7:3 ratio of slow-release fertilizer to urea is an effective fertilization strategy for achieving high yield and superior quality in rice.

Straw incorporation is an important strategy for managing crop straw resources and improving soil fertility [10]. As straw decomposes after incorporation, these nutrients are gradually released, increasing soil organic matter content, improving the soil microbial environment, and promoting nutrient cycling [11]. However, straw incorporation also exhibits an inhibitory effect during the early growth stage. Rice straw has a high C:N ratio of 60–100, whereas the optimal C/N ratio for microbial decomposition is approximately. During the initial decomposition phase, microorganisms immobilize substantial amounts of soil available nitrogen, with reported immobilization rates typically ranging from 15 to 30 kg N ha^−1^ during the first 30 days following incorporation in temperate rice systems, thereby inhibiting rice tillering and root development [12]. Studies have shown that the positive effects of straw incorporation exhibit a distinct “time-dependent” pattern. In the short term (typically the first year), nitrogen immobilization often leads to yield reductions. In contrast, long-term straw incorporation progressively enhances soil basal fertility, thereby increasing the potential for yield improvement. This temporal dynamic of “early inhibition followed by late promotion” implies that yield response to straw incorporation depends largely on the adequacy of nitrogen supply during the early incorporation period (tillering stage) [4]. When the nitrogen immobilization induced by straw incorporation is superimposed on the slow early release of slow-release fertilizer, the nitrogen deficit during tillering may be further exacerbated, which fundamentally accounts for the poor yield performance of slow-release fertilizer applied alone under straw incorporation. In contrast, the combined application of slow-release fertilizer and urea offers a viable solution. Urea supplies readily available nitrogen during tillering to alleviate the nitrogen deficiency caused by straw decomposition during tillering, thereby ensuring early tillering and root development, while slow-release fertilizer sustains nitrogen supply during the mid-to-late stages to support grain filling and quality development. This synergistic nitrogen supply model, characterized by “early compensation with available nitrogen and late guarantee with slow-release nitrogen,” theoretically enables simultaneous yield improvement and quality enhancement under straw incorporation. Nevertheless, the interactive effects of straw incorporation and slow-release fertilizer have only recently drawn research attention.

Currently, systematic evaluations of the combined application of slow-release fertilizer and urea under straw-incorporation conditions remain insufficient. Despite the demonstrated advantages of the 70% SRF + 30% urea combination under conventional conditions [13,14] and the confirmed yield-enhancing effects of straw incorporation with sulfur-coated urea (SCU) [6], three critical knowledge gaps remain regarding its application under straw incorporation in cold rice regions. First, the combined effect of SRF and straw incorporation on yield and quality remains unexplored. Specifically, when the inherently slow early N release of SRF is superimposed on the microbial N immobilization induced by straw decomposition—a dual constraint—no study has yet systematically answered how rice yield and quality respond. Second, the applicability of the optimal 7:3 ratio of slow-release fertilizer to urea under straw incorporation, as well as its underlying regulatory mechanisms affecting rice quality, remains unclear. Although some studies have revealed the mechanisms by which slow/controlled-release fertilizers improve grain quality through the coordination of carbon and nitrogen metabolism [15], systematic investigations into the key processes governing grain quality formation—including grain-filling characteristics and the activities of starch biosynthetic enzymes, such as ADP-glucose pyrophosphorylase (AGPase), soluble starch synthase (SSS), granule-bound starch synthase (GBSS), and starch branching enzyme (SBE)—remain extremely limited. Third, most existing research has been limited to either single-year trials or long-term field experiments [11], with a notable absence of direct comparisons between the first and second years of straw incorporation. Such comparisons are critical for understanding the transition from short-term inhibition to long-term promotion associated with straw incorporation.

To address these critical gaps, we conducted a two-year field experiment using the representative japonica cultivar ‘Jinongda 667’, which is a modern large-panicle variety widely adopted in Northeast China that exhibits typical sensitivity to N management and chalkiness formation, with six treatments established in a randomized complete block design. We first hypothesized that the combined application of SRF and urea at a 7:3 ratio with straw incorporation would synergistically improve rice yield and promote root development and tiller formation. Second, this combined application would sustain N availability during the mid-to-late reproductive stages by enhancing the activities of key starch biosynthetic enzymes, thereby delaying leaf senescence, improving photosynthetic capacity, and optimizing grain filling. Third, the SRF:N (7:3) + S treatment would improve grain quality, including reduced chalkiness and amylose content, and increased protein content and gel consistency. The findings of this study are expected to provide a theoretical foundation and empirical evidence for the rational utilization of one-time basal fertilization with slow-release fertilizer and straw resources in the cold rice-growing regions of Northeast China, thereby facilitating the simultaneous achievement of high yield and superior grain quality in rice.

## 2. Results

### 2.1. Rice Yield and Its Constituent Factors

Fertilizer type significantly affected grain yield, whereas straw incorporation had no significant main effect; however, their interaction was significant (Table 1). Under straw-removed conditions, the SRF:N (7:3) treatment achieved the highest two-year average yield (9966.67 kg ha^−1^), which was 7.75% higher than that of the N treatment, whereas SRF alone did not significantly increase yield. Under straw incorporation, SRF:N (7:3) + S also produced the highest yield (9633.34 kg ha^−1^), exceeding N + S by 6.64%, while SRF + S reduced yield by 7.57% relative to N + S.

Regarding the number of productive panicles, straw incorporation reduced this parameter across all treatments, although the extent of reduction varied with fertilizer type: N + S decreased by 7.42% on average compared with N, SRF + S decreased by 3.15% on average compared with SRF, and SRF:N (7:3) + S decreased by 7.90% on average compared with SRF:N (7:3). Under straw-removed conditions, the SRF:N (7:3) treatment significantly increased productive panicle number by 4.93% compared with the N treatment. The negative effect of straw incorporation on productive panicles was more pronounced in treatments receiving slow-release fertilizer. For spikelets per panicle, under straw-removed conditions, the two-year average values of spikelets per panicle were significantly higher in the SRF and SRF:N (7:3) treatments than in the N treatment, increasing by 14.46% and 11.12%, respectively. The response of spikelets per panicle to straw incorporation differed among fertilizer types. Compared with N, the N + S treatment increased spikelets per panicle by 5.48% on average, whereas the SRF + S and SRF:N (7:3) + S treatments decreased this parameter by 5.35% and 2.24%, respectively, compared with their corresponding non-straw-incorporated counterparts. Thousand-grain weight and seed setting rate exhibited limited variation across treatments and were not significantly affected by fertilizer type or straw incorporation.

**Figure 1:**
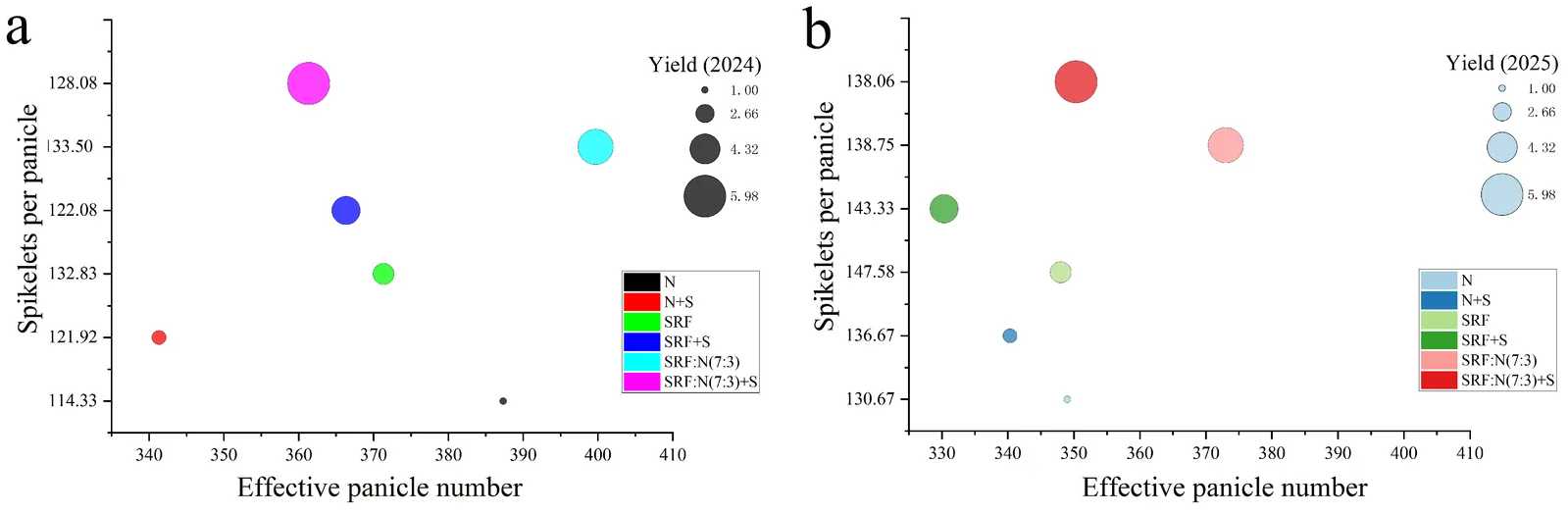
Bubble plots of yield, effective panicle number, and spikelets per panicle under different treatments in 2024 (**a**) and 2025 (**b**). The *x*-axis represents effective panicle number (×10^4^ hm^−2^), the *y*-axis represents spikelets per panicle, and bubble size represents grain yield (kg hm^−2^). N: urea; N + S: urea with straw returning; SRF: slow-release fertilizer; SRF + S: slow-release fertilizer with straw returning; SRF:N (7:3): 70% SRF blended with 30% urea; SRF:N (7:3) + S: 70% SRF blended with 30% urea with straw returning. Different colors indicate different fertilizer treatments. Data are presented as means of three replicates.

Two-way ANOVA indicated that neither the main factors nor their interaction significantly influenced 1000-grain weight or seed setting rate. To further quantify the relative contribution of each yield component to grain yield, Pearson correlation analysis was performed using treatment means across both years (*n* = 12). Grain yield was significantly and positively correlated with spikelets per panicle (r = 0.676, *p* < 0.05), whereas no significant correlation was detected between yield and productive panicle number (r = −0.065, *p* > 0.05), 1000-grain weight (r = −0.157, *p* > 0.05), or seed setting rate (r = −0.317, *p* > 0.05). These results confirm that the yield advantage of the SRF:N (7:3) treatment primarily arose from increased spikelets per panicle. The negative trend between yield and seed setting rate (r = −0.317), though not statistically significant, is consistent with the slightly lower seed setting rate observed in the highest-yielding SRF:N (7:3) treatment. The yield advantage of SRF:N (7:3) was consistently maintained across both years, as illustrated in the bubble plot (Figure 1), where this treatment consistently occupied the upper-right region with the largest bubble size, primarily driven by its higher spikelets per panicle. Notably, the yield gap between the N + S and SRF:N (7:3) + S treatments narrowed, likely due to the progressive improvement in soil fertility following two consecutive years of straw incorporation (Figure 1b).

### 2.2. Effects of Combined Application of Slow-Release Fertilizer and Urea Synergized with Straw Incorporation on Nitrogen Accumulation in Rice

Two-way ANOVA (Table S1) revealed that fertilizer type had significant effects on nitrogen accumulation at maturity, whereas straw incorporation significantly affected nitrogen accumulation. The interactive effects between fertilizer and straw incorporation on N accumulation reached significant or highly significant levels at both the full heading stage and the grain-filling stage, indicating that fertilization and straw incorporation synergistically regulated nitrogen uptake, particularly during the mid-to-late growth periods.

**Figure 2:**
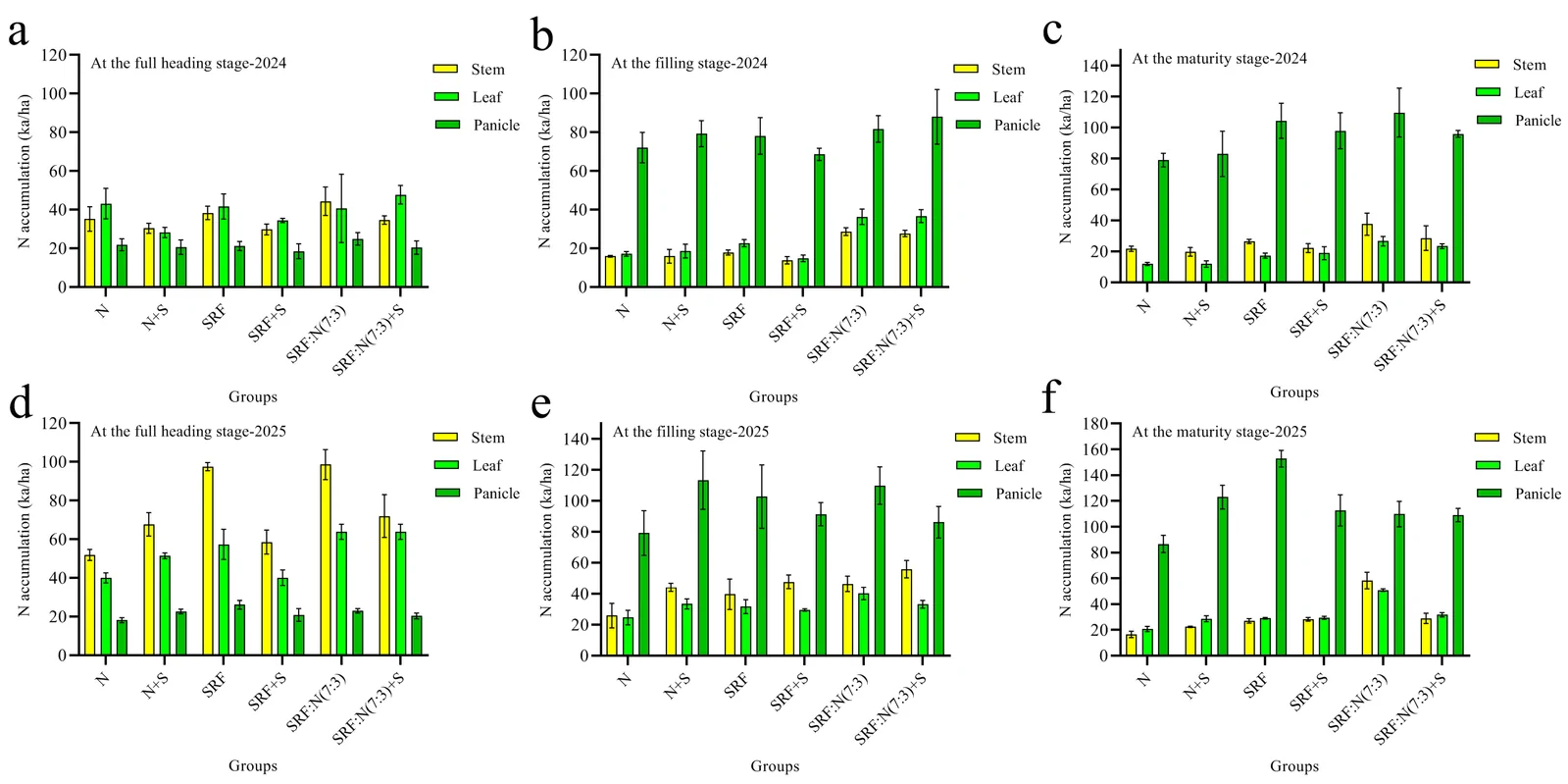
Nitrogen (2024 (**a**–**c**), 2025 (**d**–**f**)) accumulation in stems, leaves, and panicles of rice under different treatments at three growth stages in 2024 and 2025. N, urea; N + S, urea with straw incorporation; SRF, slow-release fertilizer; SRF + S, slow-release fertilizer with straw incorporation; SRF:N (7:3), 70% SRF blended with 30% urea; SRF:N (7:3) + S, 70% SRF blended with 30% urea with straw incorporation. Error bars represent the standard error (SE) of the mean (*n* = 3).

In 2024, at the full heading stage, stem N accumulation was highest in the SRF:N (7:3) treatment (44.40 kg ha^−1^), which was 26.17% higher than that in the N treatment (35.19 kg ha^−1^). In contrast, stem N accumulation under the SRF + S treatment (29.79 kg ha^−1^) was 22.36% lower than that under the SRF treatment (38.37 kg ha^−1^). Leaf N accumulation was highest under the SRF:N (7:3) + S treatment (47.74 kg ha^−1^), representing a 69.43% increase compared with the N + S treatment (28.18 kg ha^−1^). Panicle N accumulation showed relatively small differences among treatments, with the SRF:N (7:3) treatment exhibiting the highest value (24.89 kg ha^−1^) and the SRF + S treatment the lowest (18.50 kg ha^−1^) (Figure 2a). At the filling stage, stem N accumulation was highest in the SRF:N (7:3) treatment (28.66 kg ha^−1^) and lowest in the SRF + S treatment (13.84 kg ha^−1^), with the former being 107.03% higher than the latter. Leaf N accumulation was highest under the SRF:N (7:3) + S treatment (36.73 kg ha^−1^), which was 97.36% higher than that under the N + S treatment (18.61 kg ha^−1^). Panicle N accumulation was highest under the SRF:N (7:3) + S treatment (88.04 kg ha^−1^), exceeding that under the N treatment (72.13 kg ha^−1^) by 22.07% (Figure 2b). At maturity, stem N accumulation was highest in the SRF:N (7:3) treatment (37.66 kg ha^−1^), which was 73.13% higher than that in the N treatment (21.75 kg ha^−1^). Leaf N accumulation was also highest in the SRF:N (7:3) treatment (26.63 kg ha^−1^), representing a 122.80% increase over the N treatment (11.95 kg ha^−1^). Panicle N accumulation was highest in the SRF:N (7:3) treatment (109.71 kg ha^−1^), which was 38.76% higher than that in the N treatment (79.07 kg ha^−1^). Although the SRF:N (7:3) + S treatment exhibited a slightly lower panicle N accumulation at maturity (95.97 kg ha^−1^) than the SRF + S treatment (97.98 kg ha^−1^), it remained substantially higher than the N + S treatment (83.03 kg ha^−1^), representing a 15.58% increase (Figure 2c).

Across all three growth stages, N accumulation in 2025 was substantially higher than that in 2024 for most treatments and organs (Figure 2d–f), particularly at the full heading and maturity stages. For instance, at the full heading stage, stem N accumulation under the SRF:N (7:3) treatment in 2025 (98.61 kg ha^−1^) was 122.11% higher than that in 2024 (44.40 kg ha^−1^); at maturity, panicle N accumulation under the SRF treatment in 2025 (152.80 kg ha^−1^) was 46.25% higher than that in 2024 (104.47 kg ha^−1^). This inter-annual variation likely reflects differences in climatic conditions between the two growing seasons (Figure S1), which may have influenced crop N uptake and assimilation capacity. Despite the higher absolute N accumulation in 2025, the relative treatment rankings were largely consistent between the two years: SRF:N (7:3) and SRF treatments generally exhibited higher N accumulation than conventional urea treatments, whereas the SRF + S treatment frequently ranked lowest among treatments. This consistency indicates that the N supply advantage of slow-release fertilizer formulations is robust across years, although the magnitude of N accumulation may vary with seasonal conditions. Notably, the SRF:N (7:3) + S treatment maintained competitive N accumulation across both years, particularly in leaves at the full heading stage and in panicles at maturity, further supporting the synergistic effect of combined fertilizer application with straw incorporation on sustaining crop N nutrition.

### 2.3. Effects of Combined Application of Slow-Release Fertilizer and Urea Synergized with Straw Incorporation on Agronomic Traits of Rice

**Figure 3:**
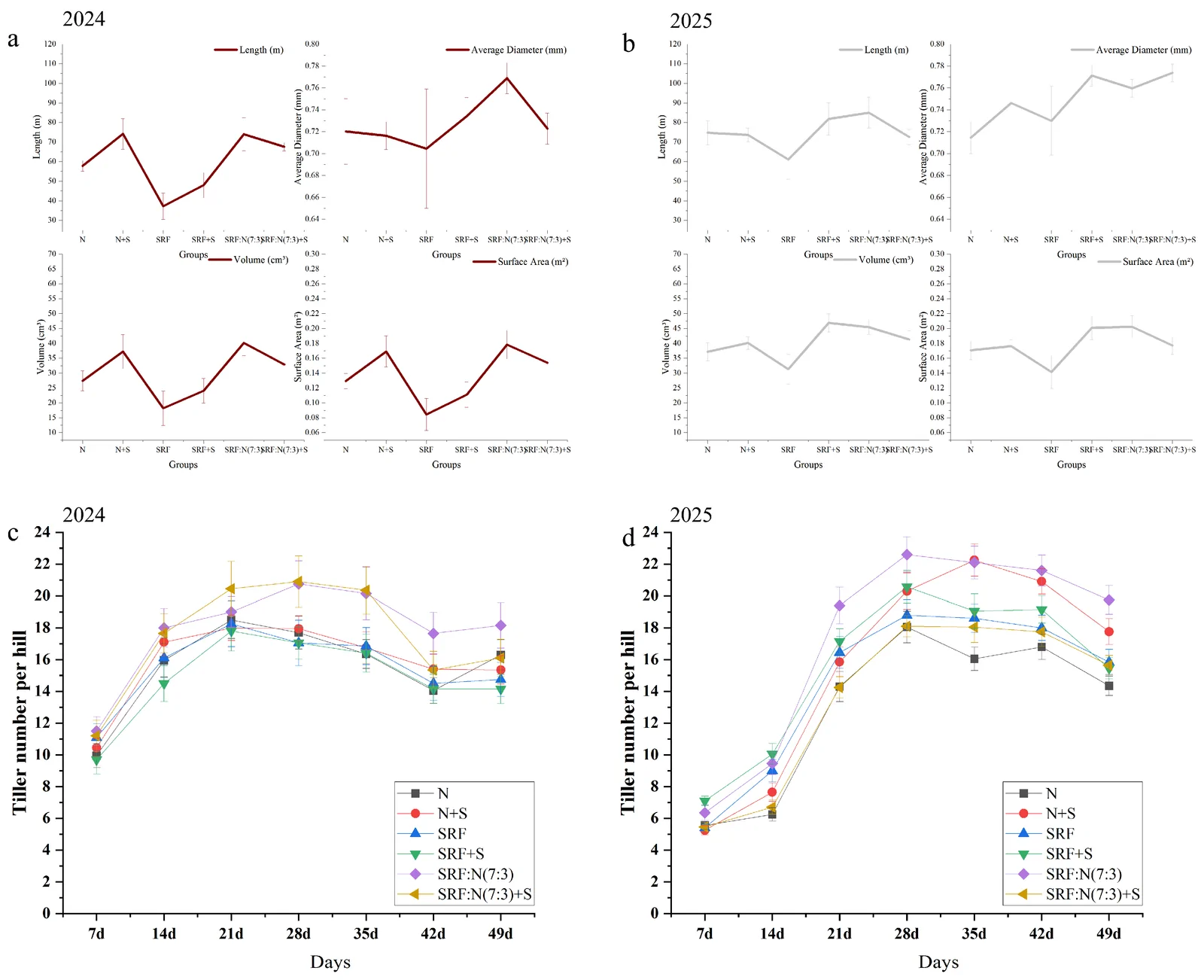
Root morphological characteristics at the tillering stage (**a**,**b**) and tillering dynamics (**c**,**d**) of rice under different treatments in 2024 and 2025. (**a**) Root length, diameter, volume, and surface area in 2024; (**b**) root traits in 2025; (**c**) tillering dynamics in 2024; (**d**) tillering dynamics in 2025. N, urea; N + S, urea with straw incorporation; SRF, slow-release fertilizer; SRF + S, slow-release fertilizer with straw incorporation; SRF:N (7:3), 70% SRF blended with 30% urea; SRF:N (7:3) + S, 70% SRF blended with 30% urea with straw incorporation. DAT, days after transplanting. Data were recorded at 7-day intervals from 7 to 49 DAT. Vertical bars indicate standard deviations (*n* = 3). Data are presented as means ± SE.

In 2024, straw incorporation significantly increased root length in the conventional fertilizer treatment (N + S), with root length being 28.41% greater than that in the N treatment. In contrast, the effect of straw incorporation was less pronounced under the slow-release fertilizer treatments, increasing root length by 28.82% in SRF + S compared with SRF, but decreasing it by 8.63% compared with SRF:N (7:3). Across fertilizer treatments, root length was shortest in the SRF treatment, being 35.59% lower than that in the N treatment. In contrast, root length in the SRF:N (7:3) treatment exceeded the N treatment by 28.02%, indicating that the addition of 30% urea effectively alleviated the inhibition of root elongation caused by slow-release fertilizer. Mean root diameter across treatments ranged from 0.72 to 0.77 mm, with no significant differences among treatments, suggesting that fertilization and straw management had limited effects on lateral root growth (Figure 3a). Compared with 2024, root length in 2025 increased by 7.37% to 70.65% across treatments (except for the N + S group), with the greatest increase observed in the SRF + S group (Figure 3b).

In 2024, the SRF:N (7:3) treatment produced the highest tiller number, peaking at 28 days after transplanting. It maintained a high tiller density of 19–20.75 tillers per hill during the 21- to 35-day period, exhibiting the longest peak-tillering duration. The SRF:N (7:3) treatment also produced the highest final tiller number among all treatments, with only a 2.60-tiller difference between the peak and final values. This demonstrates that the 7:3 combined application of slow-release fertilizer and urea effectively extended the peak-tillering period through a two-phase nitrogen supply model—”readily available nitrogen in the early stage to promote tiller initiation and slow-release nitrogen in the later stage to sustain tiller growth.” The peak tiller number in the SRF treatment (18.25) was comparable to that in the N treatment (18.50); however, the decline rate during the late tillering stage was slightly slower, indicating that the sole application of slow-release fertilizer did not confer a clear advantage during early tillering but maintained relatively stable tiller numbers due to sustained nitrogen supply (Figure 3c). Regarding straw incorporation, compared with the N treatment, the final tiller number in the N + S treatment in 2024 was slightly lower (15.35 vs. 16.30) (Figure 3c), whereas in 2025, the N + S treatment (17.76) was significantly higher than N (14.35), with an increase of 23.76% (Figure 3d). Across both years, the SRF:N (7:3) treatment consistently exhibited stable advantages in tiller initiation and productive tiller percentage. Two-way ANOVA (Table S2) showed that fertilizer treatment had a highly significant effect on final tiller number in both years, whereas straw incorporation had no significant effect in 2024 but was significant in 2025. The interaction between fertilizer and straw incorporation was not significant in either year.

### 2.4. Effects of Slow-Release Fertilizer and Straw Incorporation on Photosynthetic Parameters of Rice

The SPAD value is an important indicator reflecting relative leaf chlorophyll content and nitrogen nutritional status. Two-way ANOVA showed that fertilizer treatment significantly affected SPAD values at the jointing, full heading, and grain-filling stages. Straw incorporation significantly affected SPAD values only at the grain-filling stage, whereas the interaction between fertilizer treatment and straw incorporation was significant only at the jointing stage (Table S3). These results indicate that the regulation of leaf chlorophyll content by fertilization and straw management is primarily manifested during the mid-to-late growth stages of rice.

**Figure 4:**
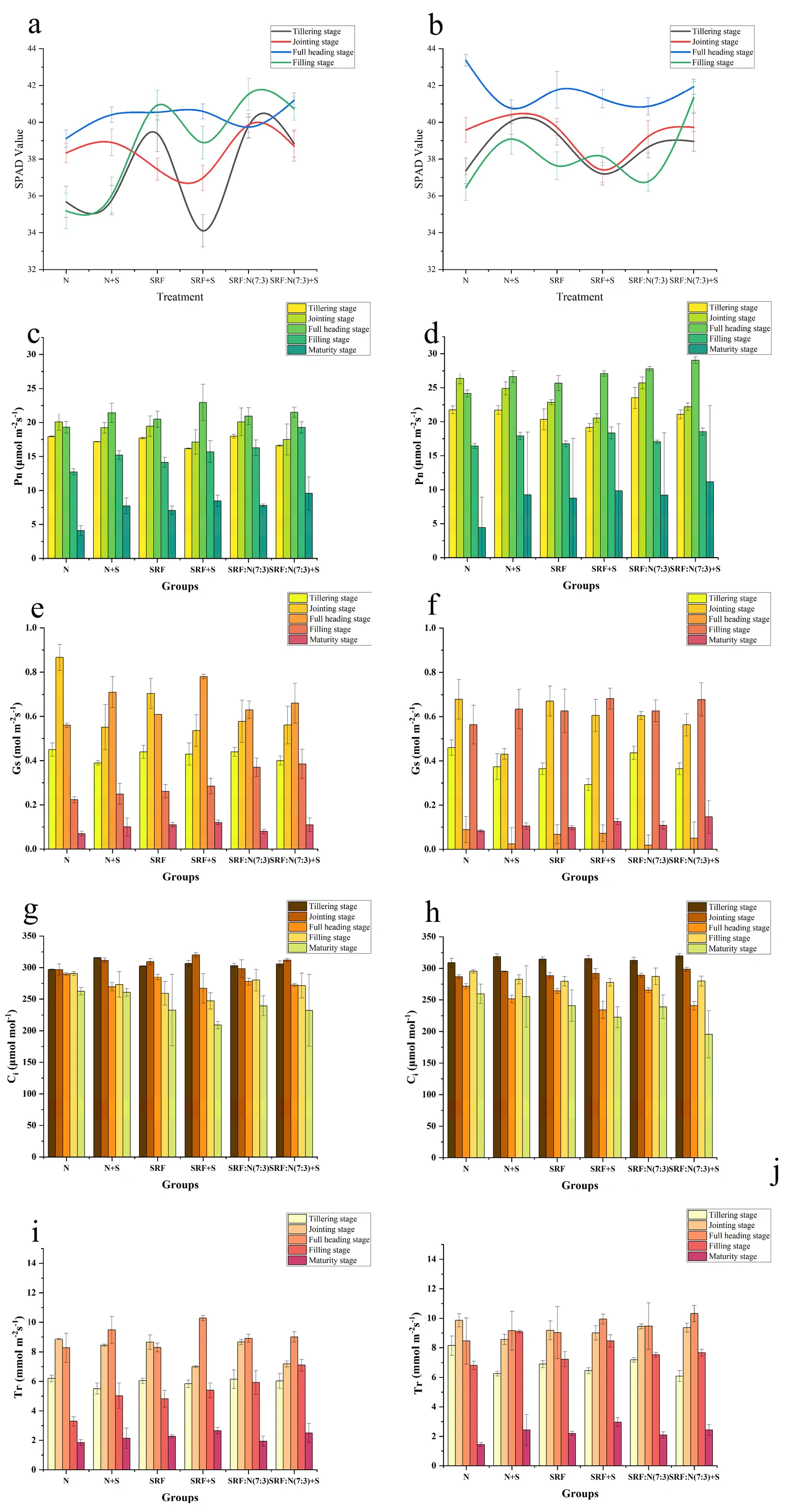
Temporal variation in leaf SPAD values (**a**,**b**) and three-dimensional response surface distributions of photosynthetic parameters (**c**–**j**) under different treatments in 2024 and 2025. (**a**) SPAD dynamics across growth stages in 2024; (**b**) SPAD dynamics across growth stages in 2025; (**c**,**d**) net photosynthetic rate (Pn); (**e**,**f**) stomatal conductance (Gs); (**g**,**h**) intercellular CO_2_ concentration (Ci); (**i**,**j**) transpiration rate (Tr). N, urea; N + S, urea with straw incorporation; SRF, slow-release fertilizer; SRF + S, slow-release fertilizer with straw incorporation; SRF:N (7:3), 70% SRF blended with 30% urea; SRF:N (7:3) + S, 70% SRF blended with 30% urea with straw incorporation. SPAD, soil–plant analysis development chlorophyll index; Pn, net photosynthetic rate; Gs, stomatal conductance; Ci, intercellular CO_2_ concentration; Tr, transpiration rate. The color gradient bar on the right corresponds to the magnitude of each parameter. Error bars represent the standard error (SE) of the mean (*n* = 3).

Across the growth period, SPAD values generally followed a unimodal pattern, increasing from tillering to a peak at the jointing to full heading stages before declining markedly during grain filling, which is consistent with leaf nitrogen remobilization and senescence processes in rice. The mean SPAD values across the entire growth period ranged from 35.18 to 41.51 in 2024 (Figure 4a) and from 36.45 to 43.37 in 2025 (Figure 4b), with generally higher levels in 2025, possibly owing to inter-annual climatic variation. At the tillering stage, SPAD values ranged from 34.10 to 39.79 in 2024 and from 37.20 to 40.06 in 2025. The SRF:N (7:3) and SRF treatments exhibited relatively high values in both years, reaching 39.79 and 39.38, respectively, in 2024, whereas the SRF + S treatment consistently recorded the lowest values (34.10 and 37.20). These results suggest that straw incorporation under SRF:N (7:3) maintained relatively strong nitrogen supply capacity during the tillering stage. The jointing to full heading stages represent the critical period of nitrogen demand in rice. At the jointing stage in 2024, the SRF:N (7:3) treatment recorded the highest SPAD value (39.86), which was 3.98% higher than that of the N treatment. In 2025, the N + S treatment recorded the highest value (40.41), although the differences among treatments were smaller than those observed at tillering. At full heading in 2024, the SRF:N (7:3) + S treatment reached the maximum value of 41.20; in 2025, the N treatment showed the highest value (43.37), while the SRF:N (7:3) + S treatment was 41.93. Notably, the N treatment exhibited a substantial decline in SPAD at grain filling in 2025, dropping to 36.45, representing a 15.95% reduction relative to full heading, whereas the SRF:N (7:3) + S treatment maintained a high value of 41.34 at grain filling. Over the two years, the SRF:N (7:3) + S treatment consistently showed significantly higher SPAD values than the N treatment at grain filling (15.72% higher in 2024 and 13.43% higher in 2025). These results indicate that the SRF:N (7:3) + S treatment sustained nitrogen supply during the late reproductive stage, thereby delaying leaf senescence and maintaining a strong photosynthetic capacity for grain filling. In contrast, although the N treatment performed relatively well at heading, its inadequate nitrogen supply during the later stages accelerated premature leaf senescence.

The mean Pn values across the entire growth period ranged from 4.10 to 22.95 μmol m^−2^ s^−1^ in 2024 (Figure 4c) and from 4.44 to 29.05 μmol m^−2^ s^−1^ in 2025 (Figure 4d), with inter-annual differences likely related to meteorological factors and measurement conditions. At the tillering stage, Pn values in 2025 were generally higher than those in 2024. In 2024, Pn at tillering ranged from 16.18 to 17.96, with relatively small differences among treatments. The SRF:N (7:3) treatment recorded the highest value (17.96) (Figure 4c). In 2025, Pn ranged from 19.15 to 23.51, with the SRF:N (7:3) treatment being significantly higher than the other treatments at 23.51, which was 22.80% higher than the lowest value observed in the SRF + S treatment (Figure 4c). These results suggest that the SRF:N (7:3) treatment enhanced early nitrogen availability and promoted early photosynthetic carbon assimilation. Notably, in 2024, the SRF + S treatment had the lowest Pn at tillering, corroborating that straw incorporation with SRF alone may limit early photosynthetic capacity due to delayed nutrient release. The jointing to full heading period represents the stage with the highest photosynthetic capacity in rice. At full heading in 2024, Pn was highest in the SRF + S treatment and lowest in the N treatment (Figure 4c). At full heading in 2025, the SRF:N (7:3) + S treatment reached a Pn of 29.05, which was significantly higher than the other treatments, being 20.20% higher than the N treatment and 13.10% higher than the SRF treatment. These results indicate that, under straw incorporation, the SRF:N (7:3) treatment maintains high carbon assimilation capacity during the critical reproductive growth stage, thereby providing sufficient photosynthates for yield formation. The grain filling to maturity period is the key window for evaluating fertilizer aftereffects. At grain filling in 2024, Pn in the SRF:N (7:3) + S treatment was as high as 19.29, which was 51.40%, 26.90%, and 36.30% higher than those in the N, N + S, and SRF treatments, respectively; at grain filling in 2025, the SRF:N (7:3) + S treatment also showed the highest Pn, being 13.0% higher than the N treatment. At maturity, Pn declined sharply across all treatments, but the SRF:N (7:3) + S treatment consistently maintained the highest values in both years, being 133.90% and 151.60% higher than the N treatment, respectively. In contrast, the conventional N treatment exhibited the greatest decline in Pn during grain filling, with maturity Pn values of only 4.10 and 4.44. This result suggests that conventional split nitrogen application failed to sustain an adequate nitrogen supply during the late reproductive stage, thereby accelerating leaf senescence and reducing photosynthetic capacity.

Changes in stomatal conductance (Gs) and transpiration rate (Tr) further supported this conclusion. Gs followed a unimodal trend over the growth period, reaching a peak from jointing to full heading (Figure 4e,f), consistent with the Pn pattern. The SRF:N (7:3) and SRF treatments exhibited higher Gs than the N treatment from full heading to maturity, but lower Gs at tillering and jointing. The dynamics of intercellular CO_2_ concentration (Ci) were not entirely consistent with Gs. At full heading in 2024, the Ci of the SRF:N (7:3) + S treatment was 272.48 μmol mol^−1^, whereas that of the SRF + S treatment was only 267.18 μmol mol^−1^. The higher Pn and Gs observed in the SRF:N (7:3) + S treatment, together with its Ci, suggest greater CO_2_ assimilation efficiency and more effective Ci utilization (Figure 4g). At grain filling in 2025, the Ci of the SRF:N (7:3) + S treatment was 279.75 μmol mol^−1^, significantly lower than that of the N treatment (Figure 4h), yet its Pn was higher than that of the N treatment. The two-year trend for Tr was similar to that of Gs (Figure 4i,j). At grain filling in 2024, the Tr of the SRF:N (7:3) + S treatment was 7.10 mmol m^−2^ s^−1^, which was 41.23% and 31.66% higher than those of the N + S and SRF + S treatments, respectively (Figure 4i). At full heading in 2025, the Tr of this treatment reached 10.32 mmol m^−2^ s^−1^, which was 12.74% higher than that of the N + S treatment (Figure 4j). The higher Tr is physiologically beneficial for transpirational cooling and mineral nutrient transport. Notably, across both years, Tr at maturity in all treatments was below 3.00 mmol m^−2^ s^−1^. However, the SRF:N (7:3) + S treatment maintained 2.43–2.50 mmol m^−2^ s^−1^, significantly outperforming the N + S treatment, suggesting delayed leaf senescence and prolonged photosynthetic activity.

Two-way ANOVA revealed that fertilizer treatment significantly affected Pn at all growth stages. Straw incorporation significantly affected Pn at the jointing and full heading stages, while the interaction between fertilizer treatment and straw incorporation was significant at full heading and maturity (Table S4). Fertilizer treatment significantly affected stomatal conductance (Gs) at tillering and full heading, whereas straw incorporation and their interaction had no significant effects at any stage (Table S5). Neither straw incorporation, slow-release fertilizer, nor their interaction significantly affected Ci (Table S6). Fertilizer treatment significantly affected transpiration rate (Tr) at tillering, straw incorporation had a highly significant effect at grain filling, and their interaction significantly affected Tr at tillering (Table S7).

### 2.5. Effects of Slow-Release Fertilizer and Straw Incorporation on Rice Grain Quality

**Figure 5:**
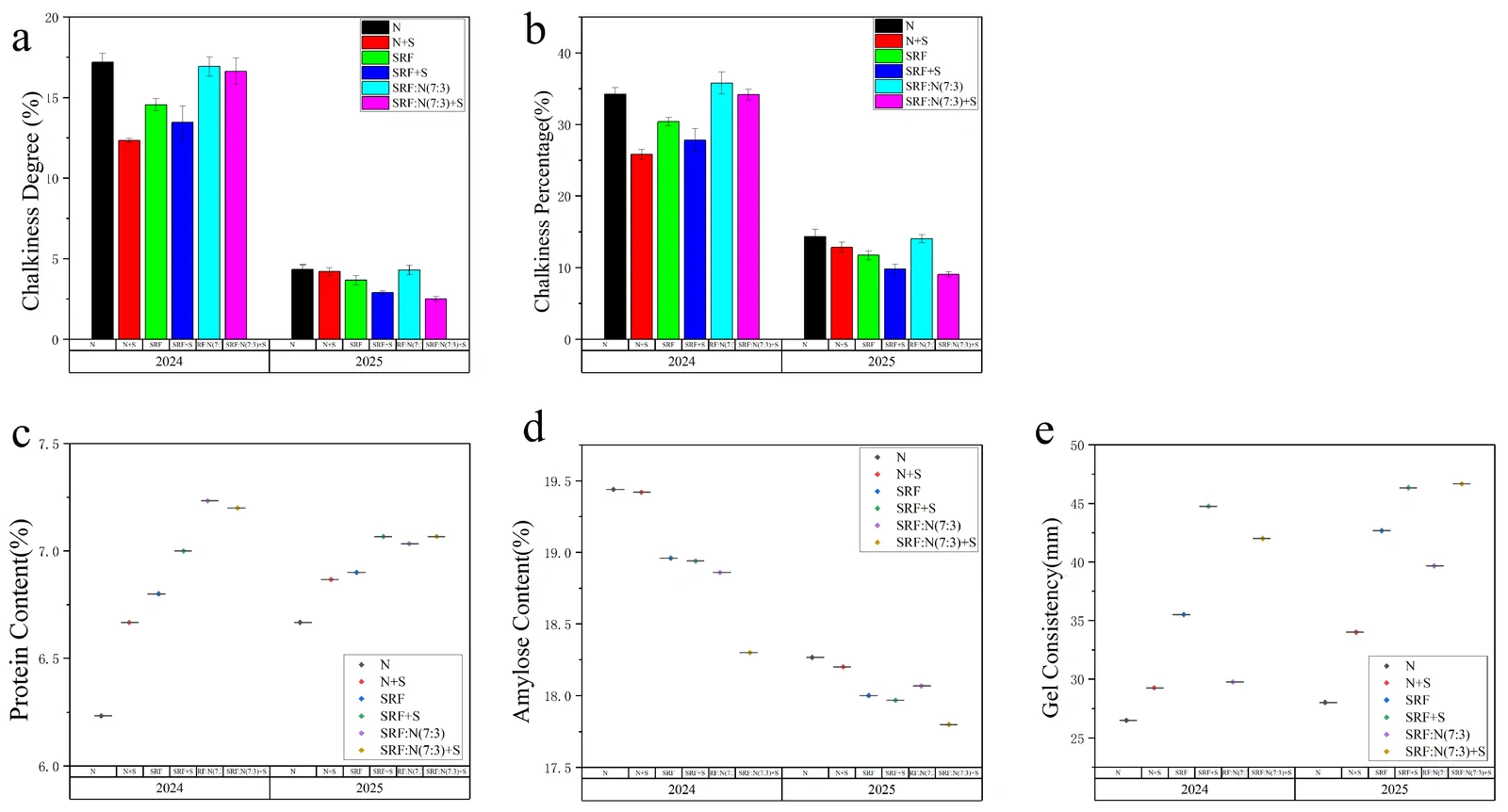
Effects of slow-release fertilizer and straw returning on grain quality traits of rice2024–2025. (**a**,**b**) Appearance quality: Chalky kernel rate and chalkiness degree; (**c**–**e**) Nutritional and eating quality: protein content, amylose content, and gel consistency; N, urea; N + S, urea with straw incorporation; SRF, slow-release fertilizer; SRF + S, slow-release fertilizer with straw incorporation; SRF:N (7:3), 70% SRF blended with 30% urea; SRF:N (7:3) + S, 70% SRF blended with 30% urea with straw incorporation. RVA, Rapid Visco Analyser. Bars represent means ± SE (*n* = 3). Different letters above bars indicate significant differences among treatments at *p* < 0.05.

Data from 2024–2025 showed that straw incorporation significantly reduced chalky grain rate and chalkiness degree across all treatments. Compared with N, the N + S treatment reduced chalky grain rate by 10.67–24.54% and chalkiness degree by 3.07–28.29%; compared with SRF, the SRF + S treatment reduced chalky grain rate by 8.55–16.48% and chalkiness degree by 7.55–20.91%; and compared with SRF:N (7:3), the SRF:N (7:3) + S treatment reduced chalky grain rate by 4.56–35.55% and chalkiness degree by 1.77–41.86%. The improvement in appearance quality resulting from straw incorporation was most pronounced in the SRF:N (7:3) treatment, indicating a synergistic effect between straw incorporation and combined fertilizer application in enhancing appearance quality (Figure 5a,b). Neither straw incorporation, fertilizer type, nor their interaction had significant effects on milling quality parameters, suggesting that milling quality is predominantly influenced by genetic and environmental factors, with limited regulatory effects from the fertilization and straw management practices employed in this study (Figure S2a–c).

Combining the two-year data, under straw-removed conditions, protein content ranked as SRF:N (7:3) > SRF > N, with SRF:N (7:3) being 5.5–16.04% higher than N. Under straw incorporation, protein content increased across all treatments: N + S increased by 3–6.95% compared with N, SRF + S increased by 2.42–2.94% compared with SRF, and SRF:N (7:3) + S increased by only 0.47% compared with SRF:N (7:3) in 2025 (Figure 5c). The sustained nitrogen supply from slow-release fertilizer promoted protein synthesis and accumulation in grains. In contrast to protein content, slow-release fertilizer treatments reduced amylose content. In 2024, under straw-removed conditions, SRF and SRF:N (7:3) decreased amylose content by 2.47% and 2.98%, respectively, compared with N. Under straw incorporation, SRF + S and SRF:N (7:3) + S reduced amylose content by 2.47% and 5.77%, respectively, compared with N + S. In 2025, under straw-removed conditions, SRF and SRF:N (7:3) decreased by 1.46% and 2.98%, respectively; under straw incorporation, SRF + S and SRF:N (7:3) + S decreased by 1.28% and 2.2%, respectively. Across both years, the SRF:N (7:3) + S treatment consistently exhibited the lowest amylose content (Figure 5d), which contributes to improved rice texture and palatability.

Gel consistency is an important indicator reflecting the softness of cooked rice, with higher values indicating softer texture. Under straw-removed conditions in 2024, the SRF and SRF:N (7:3) treatments increased gel consistency by 33.96% and 12.26%, respectively, compared with N. Under straw incorporation, SRF + S and SRF:N (7:3) + S increased gel consistency by 52.99% and 43.59%, respectively, compared with N + S. The SRF + S treatment showed the highest gel consistency, indicating that slow-release fertilizer applied alone significantly improved the softness of cooked rice. In 2025, gel consistency was generally higher across all treatments than in 2024. Compared with N, the SRF treatment increased by 52.39% and the SRF:N (7:3) treatment by 41.68%. The straw incorporation effect was also significant: SRF + S increased by 8.59% compared with SRF, SRF:N (7:3) + S increased by 17.65% compared with SRF:N (7:3), and N + S increased by 21.43% compared with N (Figure 5e). The SRF + S and SRF:N (7:3) + S treatments consistently exhibited the highest gel consistency across both years, significantly outperforming conventional fertilization treatments. Regarding RVA profile characteristics, slow-release fertilizer treatments significantly increased breakdown viscosity and decreased setback viscosity, indicating that slow-release fertilizer improved the cooking and eating quality of rice by optimizing grain starch structure. Straw incorporation further enhanced this effect, with the SRF + S and SRF:N (7:3) + S treatments showing the most pronounced improvements in increased breakdown viscosity and decreased setback viscosity (Figure S2d,e).

### 2.6. Effects of Slow-Release Fertilizer and Straw Incorporation on Grain-Filling Characteristics of Rice

**Figure 6:**
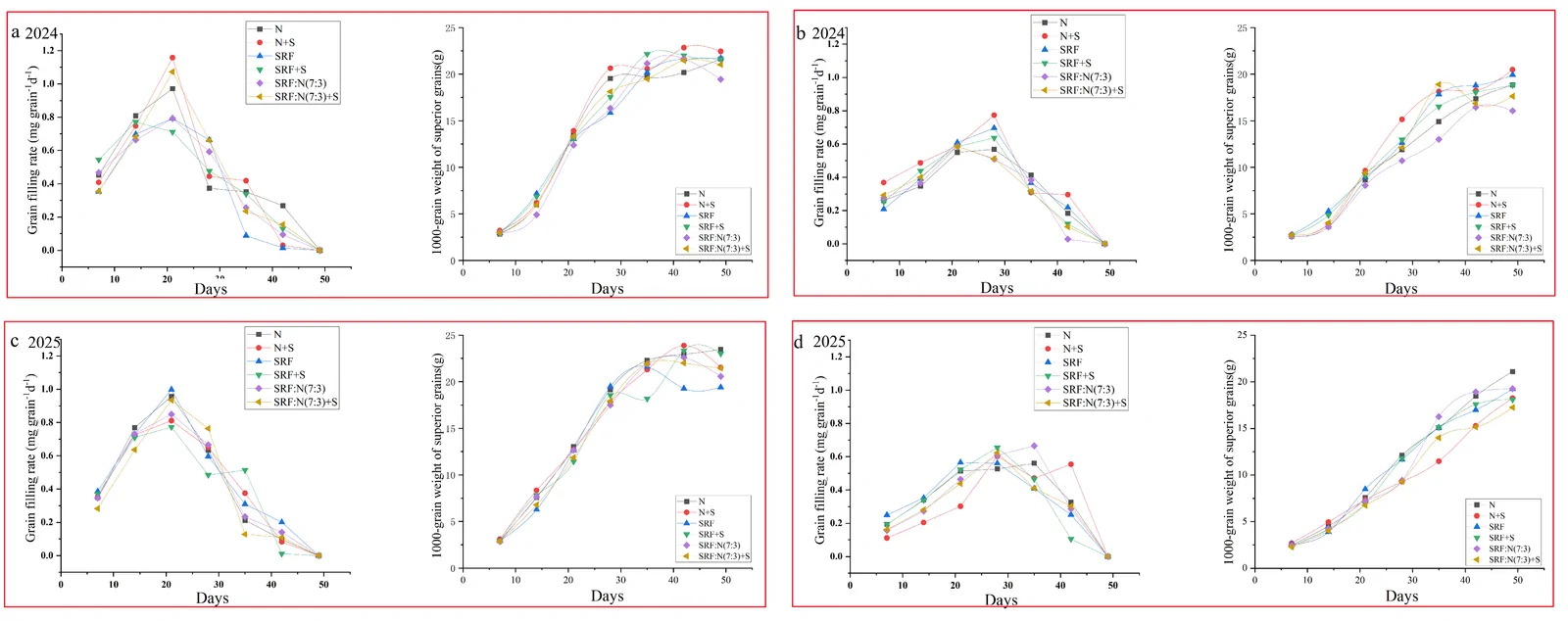
Dynamic changes of grain-filling rate and grain weight during grain filling under different treatments. (**a**) the dynamic change of strong grain grouting and grain weight in 2024; (**b**) the grouting dynamic changes and grain weight of weak grains in 2024; (**c**) dynamic change of strong grain grouting and grain weight in 2025; (**d**) dynamic changes of grain filling and grain weight of weak grains in 2025. N, urea; N + S, urea with straw incorporation; SRF, slow-release fertilizer; SRF + S, slow-release fertilizer with straw incorporation; SRF:N (7:3), 70% SRF blended with 30% urea; SRF:N (7:3) + S, 70% SRF blended with 30% urea with straw incorporation. DAF, days after flowering. Data were measured at 7-day intervals from 7 to 49 DAF. Vertical bars indicate standard deviations (*n* = 3).

The grain-filling rate of superior grains exhibited a unimodal curve, with the peak occurring at 14–21 days after flowering (DAF) (Figure 6). The effect of straw incorporation on grain-filling rate varied with nitrogen fertilizer type. The N + S treatment induced significant nitrogen immobilization during the early filling stage (7–14 DAF), resulting in reductions of 7.56% and 5.67% in the peak filling rate of superior grains in the two consecutive years, respectively, relative to the N treatment. Nevertheless, the decrease in filling rate was attenuated during the mid-to-late filling stage (after 21 DAF). The SRF + S treatment significantly increased the peak filling rate of superior grains in 2024 by 10.61% compared with the SRF treatment, but showed a reduction in 2025. This suggests that inter-annual climatic conditions (e.g., temperature and precipitation) may modulate the synergistic effects between straw decomposition and nutrient release from controlled-release fertilizer. The SRF:N (7:3) + S treatment moderately increased the peak filling rate of superior grains in both years, displaying a positive synergistic effect (Figure 6a,c). The peak filling rate of inferior grains was significantly lower than that of superior grains, with the peak delayed to 21–28 DAA. The enhancement of the peak filling rate of inferior grains by straw incorporation was only evident in the N + S treatment in 2024, whereas under most other treatments, straw incorporation exhibited inhibition or no significant effect (Figure 6b,d). These results indicate that inferior grains are more sensitive to changes in the soil micro-environment induced by straw incorporation (e.g., insufficient early nitrogen supply).

The results of 1000-grain weight at maturity showed that the enhancing effect of straw incorporation was highly dependent on nitrogen management regime and year. In 2024, for superior grains, the N + S treatment significantly increased grain weight by 3.74% compared with the N treatment. The SRF + S treatment showed little change compared with SRF, whereas the SRF:N (7:3) + S treatment substantially increased grain weight by 8.10%, indicating that the combined application of straw with slow/controlled-release and readily available nitrogen effectively compensated for the losses caused by early nitrogen immobilization (Figure 6a). It should be noted that these values represent observed trends in separately measured superior grains (Figure 6), whereas the overall 1000-grain weight based on mixed grains (Table 1) did not show significant treatment effects, reflecting the differential responses of superior versus inferior grains to N management. In 2025, the SRF:N (7:3) + S treatment increased grain weight by 4.26% compared with the SRF:N (7:3) treatment. The SRF + S treatment performed particularly well, with grain weight increasing substantially by 18.67% over SRF, which may be related to favorable climatic conditions during grain filling in 2025 that promoted straw mineralization and release. In contrast, the N + S treatment exhibited a slightly lower grain weight than the N treatment, indicating an “antagonistic effect” (Figure 6c). The performance of straw incorporation on inferior grains was highly unstable (Figure 6b,d), suggesting that in specific years or under inferior grain sink capacity limitations, straw incorporation may be detrimental to the filling of inferior grains.

### 2.7. Effects of Combined Application of Slow-Release Fertilizer and Urea Synergized with Straw Incorporation on Activities of Key Starch Biosynthetic Enzymes in Rice Grains

**Figure 7:**
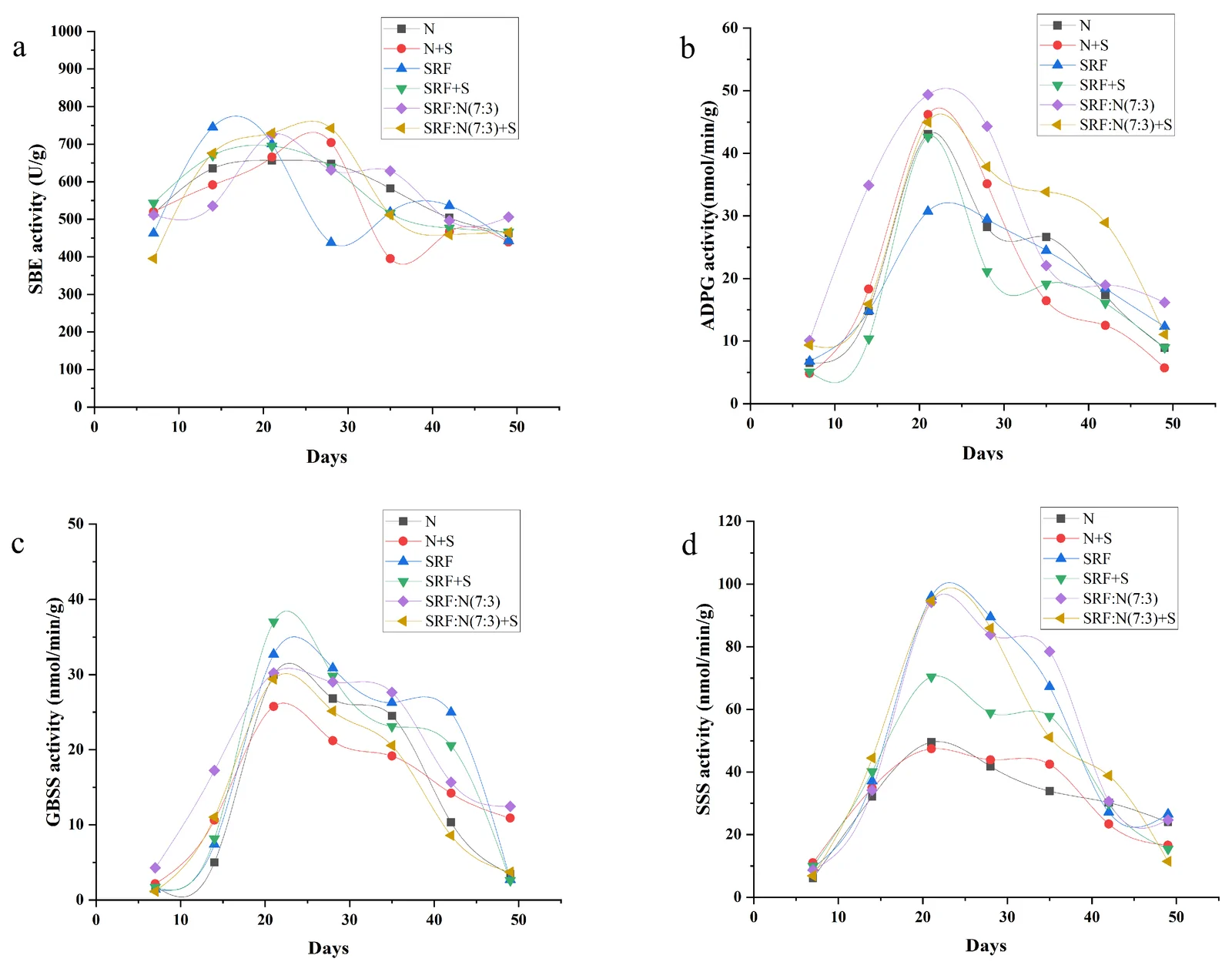
Dynamic changes in the activities of key starch synthesis enzymes in rice grains during grain filling under different treatments in 2025. (**a**) Starch branching enzyme (SBE); (**b**) ADP-glucose pyrophosphorylase (AGPase); (**c**) Granule-bound starch synthase (GBSS); (**d**) Soluble starch synthase (SSS). N, urea; N + S, urea with straw incorporation; SRF, slow-release fertilizer; SRF + S, slow-release fertilizer with straw incorporation; SRF:N (7:3), 70% SRF blended with 30% urea; SRF:N (7:3) + S, 70% SRF blended with 30% urea with straw incorporation. DAF, days after flowering. Data were measured at 7-day intervals from 7 to 49 days after flowering. Vertical bars indicate standard deviations (*n* = 3).

The activities of the four key starch biosynthetic enzymes across all treatments exhibited a unimodal trend of first increasing and then decreasing, with the progression of grain filling (Figure 7) peaking at 21 DAF and subsequently declining.

Starch branching enzyme (SBE) is a key enzyme in amylopectin synthesis. SBE activity across all treatments exhibited a unimodal temporal pattern, peaking at 21–28 DAF depending on treatment (Figure 7a). At 21 DAF, the SRF:N (7:3) + S treatment exhibited the highest SBE activity at 729.59 U/g. At 28 DAF, the SRF:N (7:3) + S treatment reached its maximum (742.17 U/g), exceeding the 21 DAF value. Compared with the N treatment, the SRF:N (7:3) treatment increased SBE activity by 10.53%, and the SRF:N (7:3) + S treatment by 11.08%, respectively, at the peak filling stage. Slow-release fertilizer treatments significantly enhanced SBE activity at the peak filling stage compared with conventional urea treatments. The effect of straw incorporation on SBE activity exhibited a stage-dependent pattern: straw incorporation increased SBE activity from 7 to 14 DAF but showed treatment-dependent effects at later stages. Specifically, at 21 DAF, N + S and SRF + S increased SBE activity by 1.35% and 0.50% compared with N and SRF, respectively, while SRF:N (7:3) + S exhibited a 0.50% increase over SRF:N (7:3). From 42 to 49 DAF, straw incorporation decreased SBE activity across all treatments (Figure 7a).

ADP-glucose pyrophosphorylase (AGPase) is the first rate-limiting enzyme in the starch biosynthesis pathway, catalyzing the formation of ADP-glucose (ADPG), and plays a critical regulatory role in starch accumulation rate. AGPase activity across all treatments peaked at 21 DAF (Figure 7b). At 21 DAF, At 21 DAF, the SRF:N (7:3) treatment exhibited the highest AGPase activity (49.37 nmol min^−1^ g^−1^), which was 14.65% higher than that of the N treatment (43.06 nmol min^−1^ g^−1^) and 6.88% higher than that of the N + S treatment (46.19 nmol min^−1^ g^−1^). From 28 to 49 DAF, enzyme activity declined rapidly across all treatments, with the N + S treatment showing the sharpest decline from 46.19 at 21 DAF to 5.72 nmol min^−1^ g^−1^ at 49 DAF. The SRF:N (7:3) and SRF:N (7:3) + S treatments maintained relatively higher AGPase activities during the late filling stage (49 DAF: 16.17 and 11.08 nmol min^−1^ g^−1^, respectively) than the N treatment.

Granule-bound starch synthase (GBSS) is primarily responsible for amylose synthesis. GBSS activity across all treatments peaked at 21 DAF (Figure 7c). At 21 DAF, the SRF + S treatment exhibited the highest GBSS activity at 37.02 nmol min^−1^ g^−1^. Straw incorporation significantly increased GBSS activity in the SRF + S treatment, but this did not result in a corresponding reduction in amylose content, suggesting that the coordination between starch synthesis and accumulation was suboptimal. From 28 to 35 DAF, the decline in GBSS activity was slowest in the SRF:N (7:3) treatment, which maintained higher activity (29.02 nmol min^−1^ g^−1^ at 28 DAF and 27.64 nmol min^−1^ g^−1^ at 35 DAF) than the N treatment (26.81 and 24.51 nmol min^−1^ g^−1^, respectively). Slow-release fertilizer treatments maintained relatively high GBSS activity during the mid-to-late filling stages. Among the slow-release fertilizer treatments, SRF and SRF:N (7:3) maintained relatively high GBSS activity during the mid-to-late filling stages (28–35 DAF), whereas SRF + S and SRF:N (7:3) + S declined to levels comparable to or below those of the N treatment by 49 DAF.

Soluble starch synthase (SSS) is the primary enzyme catalyzing the elongation of both amylose and amylopectin chains, and its activity directly affects starch synthesis rate. SSS activity across all treatments also peaked at 21 DAF (Figure 7d). At 21 DAF, the SRF treatment exhibited the highest SSS activity at 96.07 nmol min^−1^ g^−1^, followed by SRF:N (7:3) + S (94.70 nmol min^−1^ g^−1^), SRF:N (7:3) (94.15 nmol min^−1^ g^−1^), SRF + S (70.42 nmol min^−1^ g^−1^), N (49.53 nmol min^−1^ g^−1^), and N + S (47.42 nmol min^−1^ g^−1^). The SRF treatment increased SSS activity by 94.01% compared with the N treatment, while the SRF:N (7:3) + S and SRF:N (7:3) treatments showed increases of 91.17% and 90.08%, respectively. From 28 to 49 DAF, SSS activity declined more slowly in the SRF and SRF:N (7:3) treatments, with activities at 49 DAF still maintained at 26.53 and 24.69 nmol min^−1^ g^−1^, respectively, whereas the N treatment had already declined to 23.96 nmol min^−1^ g^−1^. The SRF:N (7:3) + S treatment exhibited a slower decline from 42 to 49 DAF compared with other treatments, remaining at 11.44 nmol min^−1^ g^−1^ at 49 DAF. These results indicate that slow-release fertilizer treatments effectively extended the peak period of SSS.

## 3. Discussion

### 3.1. Synergistic Effects of Slow-Release Fertilizer and Straw Incorporation: The Physiological Basis of Nitrogen Regulation for Yield Enhancement

Straw incorporation is an important agricultural practice for improving soil fertility; however, its effects on crop growth exhibit a distinct “time-dependent” pattern. During the early stage after incorporation, available nitrogen is immobilized by microorganisms, suppressing tillering, whereas in the mid-to-late stages, nutrient release promotes growth. Moreover, straw incorporation suppresses tillering during the early growth stage of rice, thereby reducing yield [14]. The results of the present study are consistent with this pattern. In 2024, straw incorporation reduced the final tiller number across all treatments, although the magnitude of the reduction varied among fertilizer treatments (Figure 3c). This “gradient difference” reveals an important pattern: slow-release fertilizer treatments, owing to their limited early nitrogen supply, are more sensitive to nitrogen competition induced by straw incorporation. In contrast, the 7:3 combination of slow-release fertilizer and urea (SRF:N (7:3)) effectively mitigated this constraint through a two-phase nitrogen supply strategy characterized by early compensation from readily available nitrogen and sustained nitrogen supply from slow-release fertilizer. The peak nitrogen release from the slow-release fertilizer during the grain-filling stage coincided with the declining mineralization rate in the later stage of straw decomposition, forming a relay nitrogen supply. Meanwhile, the sustained nitrogen supply from 70% slow-release fertilizer during the mid-to-late growth stages overlapped with the nutrients released from straw decomposition during the same period, jointly supporting photosynthetic production and grain filling [16,17]. This temporal complementarity between “fertilizer nitrogen and organic nitrogen” is the fundamental reason why the SRF:N (7:3) + S treatment maintained relatively high yield and grain quality under straw incorporation.

The central finding of this study is that the achievement of high yield and superior quality by SRF:N (7:3) under straw incorporation does not simply rely on the additive effects of nutrient supply, but rather on the precise temporal niche complementarity and functional synergy between its unique “readily available + slow-release” two-phase nitrogen supply pattern and the “early inhibition + late promotion” nitrogen release dynamics of straw decomposition, which collectively reshape the establishment and operation of the rice source–sink relationship. During this early phase, microbial immobilization of available nitrogen in the early period following straw incorporation constitutes the primary bottleneck limiting tiller development. Our data (Table 1, Figure 3) show that the sole application of slow-release fertilizer (SRF + S) exacerbates this bottleneck because its early nitrogen supply rate was insufficient to compensate for the immobilization deficit. Under straw incorporation, root length in SRF:N (7:3) + S was greater than that in SRF + S but remained lower than that in SRF:N (7:3) (without straw incorporation), indicating that the inhibitory effect of straw incorporation on root growth was only partially alleviated rather than completely eliminated under the combined application regime. These findings suggest that the readily available nitrogen (45 kg N ha^−1^) provided by the 30% urea component effectively covers the microbial immobilization gap (approximately 15–30 kg N ha^−1^) during the early phase of straw decomposition—specifically, the tillering-to-jointing stage, which is the critical window for tiller initiation and root elongation—thereby ensuring that early N supply is not constrained by straw-induced immobilization [18]. Beyond N immobilization, the initial anaerobic decomposition of rice straw in flooded paddy soils generates short-chain organic acids—including acetic, propionic, and butyric acids—that are known to be phytotoxic to rice roots. These organic acids accumulate during the early stage of straw incorporation when oxygen depletion favors anaerobic fermentation [19]. Studies have demonstrated that root elongation of rice seedlings decreases with increasing organic acid concentrations, and new root initiation can be totally inhibited at higher concentrations [20]. Moreover, the cumulative toxicity of multiple organic acids can synergistically suppress plant growth, even when each acid individually is present at non-effective concentrations. In cold-region paddy fields, straw incorporation exacerbates the reductive toxicity caused by flooding during the rice tillering stage [21]. Furthermore, organic acid accumulation in straw-amended soils has been shown to be significantly negatively correlated with rice seedling emergence, indicating that organic acid phytotoxicity is a major constraint to early crop establishment [22]. Therefore, the early-season inhibition of root growth under straw incorporation likely results from a dual constraint: (1) microbial N immobilization, which reduces available N for plant uptake, and (2) organic acid phytotoxicity, which directly damages root tissues and impairs root physiological function. In the context of our study, the 30% urea component in the SRF:N (7:3) blend may have alleviated not only the N deficit but also indirectly mitigated organic acid stress by promoting faster straw decomposition and reducing the accumulation of phytotoxic intermediates. Additionally, appropriate water management practices, such as mid-season drainage, have been shown to alleviate reductive toxicity in straw-incorporated paddies [21]. This dual perspective provides a more comprehensive understanding of the root growth inhibition observed during the early stage of straw incorporation and underscores the importance of balanced N management in mitigating both nutritional and chemical stresses.

During the heading–filling stage, source–sink relationships are optimized through a synergistic effect. During the reproductive growth stage, the advantages of slow-release fertilizer became fully apparent. At this point, the 70% slow-release fertilizer component entered its peak nutrient-release phase, which, combined with the mineralized nitrogen released from straw decomposition, generated a synergistic effect. This synergistic effect conferred two profound physiological advantages. First, it delayed functional leaf senescence and maintained high photosynthetic “source” activity. Adequate nitrogen supply effectively suppressed chlorophyll degradation in leaves during grain filling, as evidenced by the slower decline in SPAD values (Figure 4a,b) and the maintenance of higher net photosynthetic rates (Pn) (Figure 4c,d). Notably, although stomatal conductance (Gs) also increased concomitantly, intercellular CO_2_ concentration (Ci) exhibited a declining trend. This strongly demonstrates that the enhancement of photosynthetic capacity was not simply a consequence of stomatal opening, but rather a true reflection of the intrinsic activity of the photosynthetic apparatus in leaves [15], thereby providing sufficient photosynthates for grain filling. Second, it reshaped inferior grain development and unlocked the yield potential of the “sink”. In this study, the yield differences among all treatments primarily originated from variations in productive panicle number and spikelets per panicle, rather than from grain weight (Table 1). Although the SRF:N (7:3) + S treatment exhibited a reduction in productive panicle number due to straw-induced nitrogen immobilization, its spikelets per panicle declined only slightly (by 2.24%), substantially less than the 7.90% reduction in productive panicle number. Consequently, it still achieved the highest final yield (Figure 1). This finding reveals a critical “sink–source compensation” strategy whereby, with an adequate nitrogen supply during the panicle differentiation stage, facilitated by slow-release fertilizer, the plant preferentially allocated limited assimilates to superior grains, while the “synergistic nitrogen effect” during the mid-to-late filling stages significantly promoted inferior grain development (Figure 6b,d). Ultimately, the plant attained high yields through a “large panicle compensating for fewer panicles” strategy [23], reflecting a highly plastic adaptive strategy of the crop under resource-limited conditions.

Previous studies have shown that the combined application of straw incorporation with sulfur-coated urea (SCU) increased yield by 14.61–16.22% and 4.14–7.35%, respectively, compared with conventional urea combined with straw incorporation and SCU without straw incorporation, further elucidated the underlying mechanisms, showing that the combined application of straw incorporation and SCU significantly increased leaf area index, SPAD values, and net photosynthetic rate during grain filling, enhanced carbon and nitrogen metabolic enzyme activities, and increased leaf sucrose and nitrogen contents as well as plant nitrogen accumulation [6]. The physiological data from the present study are highly consistent with these findings—the SRF:N (7:3) + S treatment exhibited significantly higher SPAD values and net photosynthetic rates than the N treatment during grain filling, with even more pronounced promoting effects in the second year (2025). Notably, it has been reported that the sulfur coating of SCU is susceptible to cracking, thereby shortening the nutrient-release period [6]. This finding precisely explains the poor performance of the sole application of sulfur-coated slow-release fertilizer (SRF) in this study. Mechanical mixing and soil incorporation may have caused physical damage to the sulfur coating, resulting in a portion of the slow-release nitrogen being released prematurely, thereby further exacerbating the mismatch of relatively sufficient early nitrogen supply but insufficient mid-to-late nitrogen supply [24,25]. In contrast, the formulation adopted in this study, consisting of a 5:1 blend of sulfur- and resin-coated fertilizers, prolonged the overall release period by incorporating resin coating, thereby partially mitigating the inherent shortcomings of sulfur coating. Research on controlled-release nitrogen fertilizer (CRNF) under straw incorporation in black soil regions has further corroborated that the synergistic effects between slow-release fertilizer and straw incorporation are not only reflected in above-ground physiological traits but also in the improvement of soil fertility. That study found that CRNF combined with straw incorporation significantly increased soil organic matter, total nitrogen, and available nitrogen contents, while simultaneously reducing nitrate-nitrogen accumulation in the 30–60 cm soil layer [26]. This implies that the synergy between slow-release fertilizer and straw incorporation not only optimizes the seasonal nitrogen supply timing, but also generates a carry-over effect across growing seasons by reducing nitrogen leaching and increasing soil nitrogen pool reserves. These effects likely constitute one of the important reasons why the straw incorporation effect was more pronounced in the second year (2025) than in the first year of this study. The reversal of straw incorporation effects on tillering from inhibition in 2024 to promotion in 2025—particularly evident in the N + S treatment, which showed a 23.76% increase in final tiller number in 2025 compared with N (Figure 3d)—warrants a deeper mechanistic consideration beyond a simple “carry-over effect.” This temporal transition likely reflects progressive shifts in soil organic carbon (SOC) dynamics, nitrogen mineralization capacity, and microbial community structure under continuous straw return [11].

In the first year of straw incorporation, the input of fresh organic matter with a high C:N ratio (typically 60–100:1) creates a strong N sink, as microbial decomposers immobilize substantial amounts of available soil N to meet their stoichiometric requirements for cellulose and hemicellulose degradation. This competition for N suppresses early tiller development, as observed in 2024. However, after one year of decomposition, the residual straw-derived organic matter becomes increasingly humified, with a lower C:N ratio and reduced labile carbon fractions. Consequently, when fresh straw is incorporated in the second year, the microbial community—now adapted to a higher organic matter background—may shift from a dominance of N-immobilizing r-strategists (fast-growing, labile C consumers) toward a more balanced or even N-mineralizing K-strategists (slower-growing, recalcitrant C decomposers) [27]. This ecological succession enhances soil net N mineralization rates, as supported by studies showing that long-term straw return increases soil mineralizable N pools and microbial biomass N, with continuous straw returning increasing soil organic carbon and total nitrogen contents by 15.29% and 18.25%, respectively, while significantly enhancing urease activity [28]. Thus, by 2025, the mineralized N from both the newly incorporated straw and the residual organic matter pool likely exceeded the N immobilized during early decomposition, providing adequate available N to support tiller development and reversing the inhibitory effect observed in the first year.

This interpretation aligns with the general principle that straw incorporation effects are not static but evolve with time as the soil microbial community and organic matter fractions reach a new equilibrium. The two-year comparison in this study underscores the importance of considering temporal dynamics when evaluating the agronomic benefits of straw return, as short-term (first-year) results may underestimate its long-term potential. Under straw incorporation, slow-release fertilizer not only maintains yield but also has the potential to reduce nitrogen input, thereby providing a feasible approach for nitrogen fertilizer reduction in the black soil region of Northeast China.

### 3.2. Synergistic Regulation of Rice Grain Quality by Slow-Release Fertilizer and Straw Incorporation

Grain quality is a key determinant of the market competitiveness of rice. Previous studies have demonstrated that the type of slow/controlled-release fertilizer and fertilization regime significantly affect rice quality [29]. Chalkiness is a critical trait determining the appearance quality of rice. In the present study, straw incorporation significantly reduced chalky grain rate and chalkiness degree across all treatments (Figure 5a,b), with this effect being most pronounced in the SRF:N (7:3) treatment. This improvement is attributed to the continuously optimized nitrogen supply during grain filling, which ensured sufficient carbon and nitrogen substrates for endosperm amyloplast development, resulting in more compact starch granule packing [30]. Concurrently, improvements in RVA profile characteristics (increased breakdown viscosity and decreased setback viscosity, Figure S2d,e) and gel consistency (Figure 5c) indicated a relative increase in the proportion of amylopectin, consistent with the significant enhancement of SBE activity (Figure 7a). The reduction in amylose content and the increase in protein content significantly improved the eating and nutritional quality of rice by altering starch structure and physicochemical properties [31,32]. The results of the present study are consistent with these findings—slow-release fertilizer treatments reduced amylose content while increasing protein content, thereby improving both eating and nutritional quality of rice. Notably, the effect of straw incorporation on rice quality exhibited a “temporal cumulative effect.” In this study, straw incorporation reduced the chalky grain rate by 4.6% in 2024, and this reduction increased to 35.5% in 2025, representing an approximately eight-fold increase in the magnitude of improvement (Figure 5b). This “temporal dividend in quality improvement” may be attributable to the year-on-year enhancement of soil nitrogen supply capacity resulting from continuous straw incorporation. A three-year field experiment with controlled-release nitrogen fertilizer under straw incorporation in black soil regions demonstrated that continuous straw return increased soil organic matter and available nitrogen contents [26]. The improved soil nitrogen supply capacity, combined with the sustained nitrogen supply from slow-release fertilizer, promoted more coordinated carbon and nitrogen metabolism during grain filling, facilitated more complete amyloplast development, and resulted in a greater reduction in chalkiness in the second year. Studies on the effects of dynamic nitrogen fertilization combined with straw incorporation on rice yield and quality have shown that rice under straw incorporation exhibits lower protein content and a lower degree of chalkiness, thereby improving eating quality [33]. The essence of chalkiness formation is the loose packing of starch granules resulting from poor amyloplast development in the endosperm during grain filling [30]. The sustained nitrogen supply from slow-release fertilizer during grain-filling delays leaf senescence and ensures a continuous supply of photosynthates [34], while nutrients released during the mid-to-late stages of straw decomposition provide additional nitrogen for grain filling. Together, these processes provide sufficient carbon and nitrogen substrates for endosperm amyloplasts, resulting in more compact starch granule packing and reduced chalkiness.

The dynamics of key starch biosynthetic enzyme activities constitute the core enzymatic basis determining starch accumulation, yield, and eating quality in rice. Nitrogen fertilization management regulates starch metabolism by modulating the temporal patterns of enzyme activities during grain filling. Previous studies have shown that the novel controlled-release blended fertilizer (BBF) significantly increased the activities of ADP-glucose pyrophosphorylase (AGPase), granule-bound starch synthase (GBSS), and starch branching enzyme (SBE) during grain filling compared with conventional fertilization (CK) [15]. In addition, SRF treatment significantly enhanced the activities of AGPase, soluble starch synthase (SSS), and SBE, resulting in increases of 5.72–12.52% and 8.78–17.97% in total starch and amylopectin accumulation, respectively, while concurrently reducing GBSS activity and decreasing amylose content by 4.83–6.99% [29]. The results of the present study are consistent with these findings. The SRF and SRF:N (7:3) treatments exhibited significantly higher SSS and SBE activities at the peak filling stage than the N treatment (Figure 7a,d). These results suggest that slow-release fertilizer provides an enzymatic basis for continued starch accumulation during the late filling stage by delaying the decline in starch synthase activities. The effect of straw incorporation on enzyme activities varied markedly among enzymes and fertilizer backgrounds, with a general tendency toward suppression at the late filling stage (49 DAF) for SBE, AGPase, and SSS, regardless of fertilizer type.

An intriguing observation emerged from the comparison between GBSS activity and final amylose content. Under straw incorporation, the conventional nitrogen treatment (N + S) exhibited the lowest GBSS activity at the peak filling stage (25.76 nmol min^−1^ g^−1^) (Figure 7c), yet its amylose content remained relatively high, and the quality improvement was limited (Figure 5d,e). In contrast, the slow-release fertilizer treatments (SRF and SRF:N (7:3)) showed substantially higher GBSS activity (32.70 and 30.21 nmol min^−1^ g^−1^, respectively) at the same stage, while exhibiting significantly reduced amylose content and superior eating quality. This apparent “decoupling” between GBSS activity and amylose content suggests that the final starch composition is determined not by the peak activity of any single enzyme, but by the partitioning direction of carbon metabolic flux (ADP-glucose, ADPG) within the entire biosynthetic pathway [35]. Our data supports the following inferences. In the N + S treatment, the abundant readily available nitrogen during the early filling stage promoted vigorous protein synthesis, which may have created strong competition with starch synthesis for carbon sources. Although GBSS activity was measurable, the supply of ADPG available for amylose elongation was limited-potentially being preferentially channeled into energy metabolism associated with protein synthesis. This scenario may have resulted in low in vivo catalytic efficiency of GBSS, manifested as “low GBSS activity but not low amylose.” In the SRF treatments, the slow-release fertilizer provided a “steady and sustained” nitrogen source, thereby alleviating the intense competition between carbon and nitrogen metabolism in the early stage. This environment allowed more photosynthates (carbon flow) to enter the starch biosynthetic pathway smoothly. Under these conditions, the highly active SSS and SBE (Figure 7a,d) were more competitive in “capturing” ADPG for chain elongation and branch formation, channeling more carbon flow toward amylopectin synthesis, thereby “outcompeting” GBSS for the substrate for amylose synthesis. Beyond the regulation of starch biosynthetic enzyme activities, recent structural studies have further elucidated the relationship between starch molecular architecture and quality attributes. The formation of enzyme-resistant V-type crystalline structures in rice starch can significantly alter digestibility and physicochemical properties, with resistant starch content increasing by 12.03% upon polyphenol complexation. Although our study did not directly examine starch–polyphenol interactions, this finding highlights that the quality improvements observed under SRF treatments—particularly the reduced amylose content and optimized RVA profiles—may involve not only changes in amylose/amylopectin ratio but also alterations in starch granule crystalline organization. This structural perspective provides a complementary mechanistic layer to our enzyme activity data and suggests that future investigations should explore whether SRF-mediated nitrogen supply influences starch crystalline structure beyond simple changes in amylose content [36]. Consequently, although GBSS activity was higher under the SRF and SRF:N (7:3) treatments, the greater availability of ADPG and the competitive advantage of SSS and SBE in capturing this substrate may have redirected carbon flow toward amylopectin synthesis. This resulted in lower final amylose content, demonstrating that the direction of carbon partitioning, rather than individual enzyme peak activity, governs grain quality outcomes. This interpretation is further supported by the observed negative correlation between protein content and amylose content (Figure 5c,d), suggesting that protein-starch competition for carbon skeletons may also contribute to the decoupling phenomenon. Our finding that the SRF:N (7:3) treatment enhanced SSS and SBE activities while reducing GBSS activity is consistent with a recent report showing that slow-release nitrogen fertilizer increased AGPase, SSS, and SBE activities by 5.72–12.52% while decreasing GBSS activity by 4.83–6.99% [29].

In addition to substrate competition, protein–starch interactions may constitute another important physicochemical mechanism underlying this “decoupling”. Under slow-release fertilizer treatments, increased grain protein content may, during amyloplast development, physically embed into or coat the surface of starch granules, or alter the ionic strength and pH of the amyloplast microenvironment, thereby restricting amylose crystallization and molecular ordering. This hypothesis extends the relationship between protein and starch beyond that of “parallel indicators” to “functionally linked indicators,” suggesting a functional linkage between these components in regulating grain quality. This concept provides a potential framework for understanding nitrogen-mediated regulation of grain quality and warrants further validation through proteomic approaches and in situ characterization techniques of starch granules. The improved RVA pasting profiles and gel consistency observed under SRF treatments also carry implications for starch digestibility and nutritional quality. It has recently been reported that low-molecular-weight ferulic oligosaccharides derived from rice bran enhance the inhibitory effect on rice starch digestion by attenuating self-aggregation, suggesting that the structural reorganization of starch components—including the relative proportions of amylose and amylopectin—can profoundly influence both pasting behavior and enzymatic hydrolysis kinetics [37]. Our finding that SRF treatments increased breakdown viscosity while decreasing setback viscosity (Figure S2d,e) is consistent with a starch matrix that is more susceptible to enzymatic hydrolysis, which may translate to improved glycemic properties. This connection between agronomic nitrogen management and starch nutritional functionality represents an underexplored research avenue that warrants further investigation.

**Figure 8:**
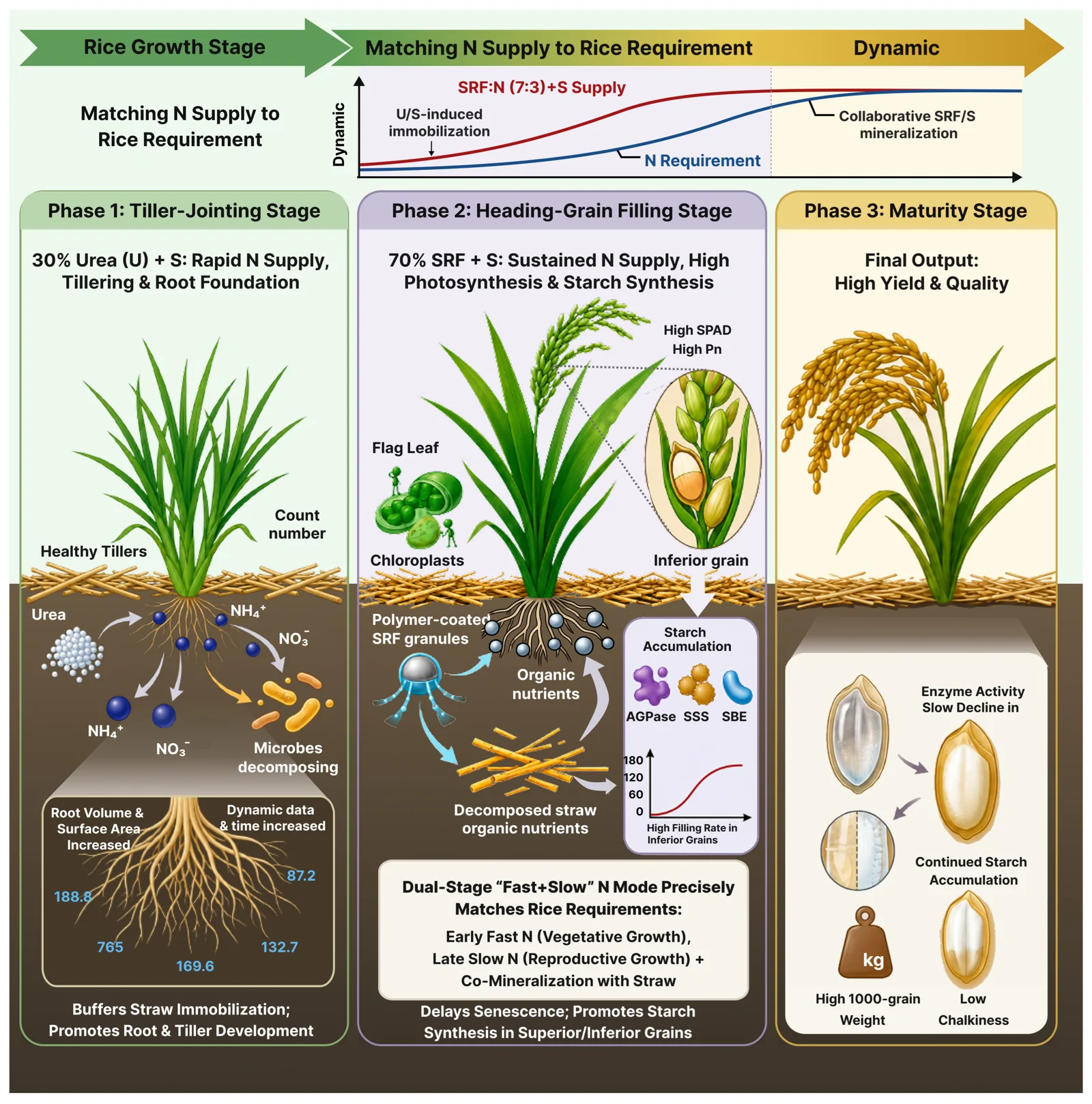
A schematic model illustrating the physiological mechanisms by which combined application of slow-release fertilizer and urea (7:3) with straw incorporation regulates grain yield and quality in rice. The diagram depicts the temporal sequence of key physiological events under the SRF:N (7:3) + S treatment from tillering to maturity. Stage I (tillering to elongation): 30% urea supplies readily available nitrogen, alleviating the dual nitrogen deficit caused by slow-release fertilizer’s initial lag and microbial immobilization during straw decomposition, thereby promoting root development and tiller formation. Stage II (full heading to grain filling): 70% slow-release fertilizer sustains nitrogen availability, maintaining leaf photosynthetic capacity, enhancing the activities of starch-synthesizing enzymes (AGPase, SSS, SBE), and improving grain filling of inferior spikelets. Stage III (maturity): delayed decline in enzyme activities during late grain filling contributes to increased 1000-grain weight and reduced chalkiness, achieving simultaneous improvement in yield and quality. The core of this model lies in the synchronization of a “readily available + slow-release” dual-phase nitrogen supply pattern with the “initial immobilization and later mineralization” temporal dynamics of straw decomposition.

Synthesizing the above findings, we propose a theoretical model of “temporal nitrogen supply driving source–sink interactions and carbon partitioning” (Figure 8). This model transcends the conventional “nutrient supply” mindset and emphasizes three key principles. First, synchrony outweighs quantity: the degree of coupling between the temporal dynamics of nitrogen supply and those of rice demand is the core determinant of yield and quality, and is more important than the total nitrogen application rate. Second, compensation precedes superposition: in straw-incorporated systems, the initial nitrogen immobilization effect must first be “offset” by readily available nitrogen to enable the subsequent “superposition” of slow-release fertilizer. Third, partitioning outweighs enzyme peaks: final grain quality is governed by the balance of carbon and nitrogen flux allocation among competing biosynthetic pathways, and the peak activity of a single enzyme alone cannot predict quality outcomes. Through a two-year field experiment, this study systematically elucidated the regulatory effects and underlying physiological mechanisms of the 7:3 combined application of slow-release fertilizer and urea synergized with straw incorporation on rice yield and quality, providing a theoretical basis for fertilization management in the cold rice region of Northeast China. Meanwhile, several unresolved limitations of this study warrant further investigation, including the hourly release dynamics of slow-release fertilizer under actual field temperature conditions, the stress effects of organic acids produced by straw decomposition on root systems and their interactions with nitrogen supply, as well as the molecular regulatory mechanisms underlying the decoupling between GBSS activity and amylose content. Future studies integrating temperature-controlled pot experiments, transcriptomic profiling, and in situ starch granule characterization are needed to provide a systems-level theoretical framework for precise regulation of rice grain quality.

From an economic perspective, although slow-release fertilizer (SRF) has a higher unit price than conventional urea, the one-time basal application of the SRF:N (7:3) blend eliminates the need for tillering and panicle top-dressings, thereby substantially reducing labor costs. In Northeast China’s rice production systems, conventional urea requires 2–3 split applications (basal + tillering + panicle fertilizer), with each top-dressing operation costing approximately 150–200 CNY ha^−1^ in labor [7]. The blended SRF:N (7:3) strategy reduces fertilization events from 3 to 1, saving an estimated 300–400 CNY ha^−1^ in labor costs. Previous studies have also reported that one-time application of blended controlled-release nitrogen fertilizer reduced labor costs by 1800 CNY ha^−1^ and enhanced economic gains by 21.5–68.8% [38]. These economic considerations, combined with the yield and quality advantages demonstrated in this study, support the practical feasibility of the SRF:N (7:3) one-time fertilization strategy for growers in cold rice regions.

## 4. Materials and Methods

### 4.1. Study Site

The field experiments were conducted from 2024 to 2025 at the National Crop Variety Approval and Characterization Station of Jilin Agricultural University, located in Changchun City, Jilin Province, China (125°24′ E, 43°48′ N). The site is situated in the Songliao Plain, the hinterland of the Northeast Plain, and has a temperate continental humid climate. The accumulated temperature and precipitation during the rice growing seasons in 2024 and 2025 are presented in Figure S1. The soil type at the experimental site is meadow black soil, with the basic physicochemical properties of the 0–20 cm topsoil layer as follows: organic matter content 17.45 g kg^−1^, alkaline hydrolyzable nitrogen 33.29 mg kg^−1^, available potassium 135.09 mg kg^−1^, available phosphorus 29.02 mg kg^−1^, and pH 6.70.

### 4.2. Experimental Design and Agricultural Practices

This experiment is a pool planting experiment. The rice cultivar used was Jinongda 667 (China Rice Data Center, No. 20243224). Six treatments were established: (1) 150 N urea without straw incorporation (N) was applied in a 6:3:1 ratio of base fertilizer: tillering fertilizer: ear fertilizer; (2) 150 N urea with straw incorporation (N + S) was applied in a 6:3:1 ratio of base fertilizer: tillering fertilizer: ear fertilizer; (3) 150 N slow-release fertilizer without straw incorporation (SRF) was applied in a single basal application; (4) 150 N slow-release fertilizer with straw incorporation (SRF + S) was applied as a single basal dose; (5) 150 N slow-release fertilizer:urea = 7:3 without straw incorporation [SRF:N (7:3)] was applied in a single basal application; and (6) 150 N slow-release fertilizer:urea = 7:3 with straw incorporation [SRF:N (7:3) + S] was applied in a single basal application (SRF refers to a 5:1 physical blend of sulfur-coated and resin-coated urea, with the coating layers serving as the primary diffusion barrier for N release). For each treatment, 150 kg/ha of pure nitrogen, 75 kg/ha of P_2_O_5_ and 75 kg/ha of K_2_O were applied, respectively; phosphorus and potassium fertilizers were applied as base fertilizers at one time. In the straw-incorporated treatments, chopped straw was uniformly spread over the plots and incorporated into the 0–20 cm soil layer using a rotary tiller before the growing season. The nitrogen fertilizers used were urea (46% N) and slow-release fertilizer, which consisted of a 5:1 blend of sulfur-coated and resin-coated urea (25% N); the phosphorus and potassium fertilizers were single superphosphate (12% P_2_O_5_) and potassium chloride (60% K_2_O), respectively. The amount of straw incorporated was 4500 kg ha^−1^. In 2024, seedlings were raised on 6 April and transplanted manually on 28 May, with a leaf age of three leaves and one growing point (3-leaf and 1-heart stage), five seedlings per hill, and harvested on 23 September. In 2025, seedlings were raised on 3 April and transplanted manually on 23 May, with a leaf age of three leaves and one growing point, five seedlings per hill, and harvested on 22 September.

Throughout the rice growing season, continuous flooding irrigation was maintained with a water depth of 3–5 cm from transplanting to 7 days before harvest. The field was drained naturally at the maturity stage to facilitate harvest. No mid-season drainage or intermittent irrigation was applied during the tillering or panicle differentiation stages. Water depth was monitored daily using a graduated staff gauge installed in each concrete pool and maintained at the target depth by periodic replenishment.

The chemical composition of the incorporated rice straw was not measured in this study. According to typical values reported in the literature for rice straw, the total carbon content ranges from 38% to 45%, total nitrogen from 0.4% to 0.8%, and the C/N ratio from 50 to 70. The lignocellulosic composition typically comprises 30–45% cellulose, 20–35% hemicellulose, and 10–25% lignin. These values were used to contextualize the microbial N immobilization dynamics in the discussion [39,40,41,42].

### 4.3. Measurement of Yield and Yield Components

At the maturity stage, three points in the middle of each plot were selected, and five representative plants at each point were selected to determine the number of grains per ear, seed setting rate, 1000-grain weight and other yield components; the theoretical yield was calculated, and the number of effective panicles within 1 m^2^ in the field was determined. The yield of all rice ears in the harvest plot was measured, after air drying and threshing, the impurities were removed; the moisture content was then weighed and measured, and the standard grain yield of each treatment was determined based on the moisture content of 14%. Each measurement was replicated three times, and the average values were calculated.

### 4.4. Nutrient Uptake

At the tillering, jointing, full heading, grain filling, and maturity stages, three representative hills with uniform growth were selected from each plot. The sampled plant organs were oven dried, ground using a sample mill, and passed through a 0.50 mm sieve. An analytical balance with 0.0001 g precision was used to accurately weigh 0.50 g of the ground sample, which was then placed at the bottom of a digestion tube, followed by the addition of 5 mL of concentrated sulfuric acid, and allowed to stand overnight. Plant organ samples were digested using the H_2_SO_4_-H_2_O_2_ digestion method [43], and the nitrogen content in the digested solutions was determined using an automatic Kjeldahl apparatus (FOSS Kjeltec™ 8400, FOSS Analytical A/S, Hillerød, Denmark). The uptake amounts of nitrogen were obtained by multiplying the nutrient content by the dry matter weight of the respective plant organs (grains and shoots).

### 4.5. Determination of Agronomic Traits

Three sampling points were selected in each plot, and at each point, 10 consecutive hills were designated for tiller dynamic monitoring. Observations began at 15 days after transplanting and were conducted at 7-day intervals until the heading stage. At the tillering stage, root samples were collected from the 0–30 cm soil layer, carefully washed, and analyzed for root length, mean diameter, root volume, and root surface area using the root analysis system software WinRhizo PRO 2016 (Regent Instruments, Québec City, QC, Canada).

### 4.6. Photosynthetic Parameter Determination

Five plants with uniform growth were selected for each measurement. SPAD values were determined 7 days after flowering (DAF) using a chlorophyll meter (SPAD-502plus, Konica Minolta, Tokyo, Japan) on the upper, middle, and lower portions of the topmost fully expanded leaf, with three replicates per leaf portion, and the average value was calculated. For photosynthetic gas exchange parameters, five representative plants were selected and measured at 7-day intervals after anthesis. Measurements were conducted on the topmost fully expanded leaf between 9:00 and 11:00 a.m. on clear, cloudless days using a Li-6400 portable photosynthesis system (LI-COR Biosciences, Lincoln, NE, USA). The leaf chamber temperature was set at 25 °C, and the photosynthetically active radiation (PAR) was maintained at 1200 μmol m^−2^ s^−1^ using the built-in LED light source. Parameters recorded included net photosynthetic rate (Pn), stomatal conductance (Gs), intercellular CO_2_ concentration (Ci) and transpiration rate (Tr). Three replicate measurements were performed per treatment, with a total of 15 leaves measured, and the mean values were calculated.

### 4.7. Grain Quality

Milling quality and appearance quality were determined according to the Chinese National Standard GB/T 1354-2018 for rice, including brown rice rate, milled rice rate, head rice rate, chalky grain rate, and chalkiness degree. The Wanshen Rice Appearance Analysis System (HNSA-YQ-048) (Hangzhou Wanshen Detection Technology Co., Ltd., Hangzhou, China) is used to accurately assess the whole milling rate and appearance quality of rice, covering the determination of key indicators, such as the chalky grain rate, degree of chalkiness and aspect ratio. The palatability quality (protein content, amylose content and palatability value) was determined by a rice palatability meter (JSWL, Beijing Dongfu Jiuheng Instrument Technology Co., Ltd., Beijing, China). For gel consistency, precisely 100 mg of rice flour sample was placed in a test tube, to which 0.2 mL of 0.025% thymol blue ethanol solution was added, and the tube was gently shaken. Subsequently, 2.0 mL of 0.2 mol L^−1^ potassium hydroxide solution was added, and the tube was shaken to fully mix the rice flour. Gel consistency was then determined following the Chinese National Standard GB/T 22294-2008. Starch pasting properties (RVA profile characteristics) of rice flour were determined using a Rapid Visco Analyser (RVA-TecMaster, Perten Instruments, Macquarie Park, Australia), with three replicates per sample, and the average values were calculated.

### 4.8. Determination of Grain-Filling Characteristics and Activities of Key Starch Biosynthetic Enzymes

Sampling commenced at 7 DAF and was conducted at 7-day intervals thereafter. At each sampling time, 10 marked hills were collected from each plot. The samples were oven-killed at 105 °C for 30 min and then dried at 80 °C to constant weight. Superior and inferior grains were separated, weighed for dry matter, and the number of grains was recorded. Grain filling was fitted using the Richards growth equation, and the grain-filling rates of superior and inferior grains were calculated accordingly. Grain-filling parameters were then computed based on the following equations [44]:

$$W\;=\;A/{\left(1\;+\;Be^{-\mathrm{Kt}}\right)}^{\frac{1}{N}}$$

$$G=\frac{\mathrm{AKB}e^{-\mathrm{Kt}}}{N{\left(1+Be^{-\mathrm{Kt}}\right)}^{\frac{N+1}{N}}}$$

In the equation, W represents grain weight (mg), A is final grain weight, and *t* is days after anthesis. B, K, and N are parameters estimated by the regression equation, and G denotes the grain-filling rate.

Superior and inferior grains were distinguished based on both panicle position and flowering sequence. On the day of heading (when the panicle neck fully emerged from the leaf sheath), panicles with uniform flowering were selected and tagged, and the date of heading was recorded as 0 days after heading (DAH). The flowering date of individual grains was recorded daily during the flowering period. Superior grains were defined as the first and second grains on the primary branches located in the middle-upper portion of the panicle, which flowered within 0–2 DAH (i.e., the earliest-flowering grains). Inferior grains were defined as grains on the secondary branches (excluding the apical grains) of the lower portion of the panicle, which flowered between 5 and 7 DAH (i.e., the latest-flowering grains). At each sampling time point, superior grains were collected from the primary branches of the middle-upper portion, while inferior grains were collected from the secondary branches of the lower portion of the tagged panicles.

Determination of key starch biosynthetic enzyme activities in grains. At the heading stage, 100 single stems with uniform growth were selected from each treatment, tagged, and the heading date was recorded. At 7, 14, 21, 28, 35, 42, and 49 days after flowering (DAF), 10 tagged single stems were collected from each treatment, and grains from the middle part of the panicles were excised, immediately frozen in liquid nitrogen for 30 s, and then stored at −80 °C for subsequent analysis. The activities of ADP-glucose pyrophosphorylase (AGPase), soluble starch synthase (SSS), granule-bound starch synthase (GBSS), and starch branching enzyme (SBE) were measured, and the assay kits for enzyme activity determination were purchased from Suzhou Grace Biotechnology Co., Ltd., Suzhou, China.

### 4.9. Data Analysis

Data organization and processing were performed using Microsoft Excel 2021. Graphs were generated using GraphPad Prism 9.5.1, the online tool ChiPlot (https://www.chiplot.online/, accessed on 20 July 2026) and Figdraw (https://www.figdraw.com/#/, accessed on 20 July 2026). Statistical analyses were carried out using IBM SPSS Statistics 26. The significance levels for all statistical tests were designated as follows: 0.01 < *p* ≤ 0.05 = *, 0.001 < *p* ≤ 0.01 = **. Prior to all ANOVA analyses, the assumptions of normality and homogeneity of variances were verified. Normality of residuals was assessed using the Shapiro–Wilk test, and homogeneity of variances was assessed using Levene’s test. In all cases, the assumptions were met (*p* > 0.05). Data from 2024 and 2025 were analyzed separately due to significant inter-annual climatic differences (Figure S1), and the consistency of treatment effects across years was confirmed by the absence of significant treatment × year interactions for the key variables. When significant differences were detected, means were compared using the least significant difference (LSD) test at the 0.05 probability level.

## 5. Conclusions

This study demonstrates that the 7:3 combined application of slow-release fertilizer and urea provides a robust strategy for achieving high yield and superior grain quality in the cold rice-growing region of Northeast China. This nitrogen supply pattern, in which readily available nitrogen supports tillering in the early stage and slow-release nitrogen ensures grain filling in the later stage, precisely matches the “early inhibition followed by late promotion” nitrogen release dynamics of straw incorporation. This combined application significantly increased productive panicle number and spikelets per panicle, maintaining yield superiority through “large panicles compensating for fewer panicles.” The two-year experimental results confirmed that the yield and quality advantages of this strategy were maintained under both straw-incorporation and straw-removal conditions. However, it should be noted that the underlying mechanisms differed between the two straw management regimes: under straw-removal conditions, yield was achieved through synergistic enhancement of both productive panicle number and spikelets per panicle, whereas under straw incorporation, yield was maintained primarily through compensation by increased spikelets per panicle, which offset the reduction in productive panicle number (approximately 7.9%) caused by early-season N immobilization. The benefits of straw incorporation became more pronounced with successive years of implementation. This study provides a theoretical basis for the spatiotemporal management of nitrogen in single basal fertilization under straw-incorporated conditions.

## Figures and Tables

**Table 1:** Effects of slow-release fertilizer and straw returning on rice yield and its components.

Year	Treatment	Effective Panicle Number (×10^4^ ha)	Spikelets per Panicle	1000-Grain Weight (g)	Seed Setting Rate (%)	Yield (kg/ha)
2024	N	387.33 ± 9.29	114.33 ± 6.38	21.84 ± 1.19	97.80 ± 0.14	8766.67 ± 665.83 ab
	N + S	341.33 ± 21.39	121.92 ± 5.20	22.31 ± 0.52	98.40 ± 0.33	8400.00 ± 556.78 b
	SRF	371.33 ± 24.83	132.83 ± 16.23	22.65 ± 1.49	97.42 ± 0.59	8533.33 ± 321.46 b
	SRF + S	366.33 ± 42.72	122.08 ± 4.19	22.32 ± 0.24	96.86 ± 0.83	7500.00 ± 655.74 c
	SRF:N (7:3)	399.67 ± 24.01	133.50 ± 8.02	21.84 ± 1.51	94.37 ± 1.36	9566.67 ± 378.59 a
	SRF:N (7:3) + S	361.33 ± 38.59	128.08 ± 7.35	21.70 ± 0.69	91.31 ± 5.04	9500.00 ± 200.00 a
2025	N	349.00 ± 9.00	130.67 ± 17.03	22.60 ± 0.56	95.57 ± 0.89	9733.33 ± 305.51 b
	N + S	340.33 ± 14.36	136.67 ± 12.69	22.63 ± 1.94	96.70 ± 3.15	9666.67 ± 416.33 b
	SRF	348.00 ± 23.00	147.58 ± 8.59	22.58 ± 0.42	96.22 ± 1.15	9700.00 ± 264.58 b
	SRF + S	330.33 ± 22.50	143.33 ± 11.69	20.89 ± 1.31	91.22 ± 6.86	9200.00 ± 200.00 b
	SRF:N (7:3)	373.00 ± 38.00	138.75 ± 12.00	21.37 ± 0.57	96.70 ± 1.87	10,366.67 ± 513.16 a
	SRF:N (7:3) + S	350.33 ± 5.03	138.06 ± 4.97	22.66 ± 0.20	95.30 ± 2.02	9766.67 ± 208.17 ab
	S	ns	ns	ns	**	ns
	F	ns	ns	ns	ns	*
	S × F	ns	ns	ns	ns	*

Note: N: Conventional fertilizer (urea); N + S: Conventional fertilizer + straw return; SRF: Slow-release fertilizers; SRF + S: Slow-release fertilizers + straw return; SRF:N (7:3): Combined application of slow-release fertilizer and urea (7:3); SRF:N (7:3) + S: Combined application of slow-release fertilizer and urea (7:3) + straw return; S: Straw return; F: Different fertilizers. S × F, interaction between straw incorporation and fertilizer treatment. Different letters in the table indicate significant differences among treatments (*p* < 0.05). An asterisk (*) denotes *p* < 0.05, double asterisks (**) denote *p* < 0.01, and “ns” indicates no significant difference, the same below.

## Data Availability

The data presented in this study are available within the article. Additional data supporting the findings of this study are available from the corresponding author upon reasonable request.

## Supplementary Materials

The following supporting information can be downloaded at: https://www.mdpi.com/article/10.3390/plants15172591/s1, Figure S1: The air temperature and daily precipitation during the rice-growing seasons from 2024 to 2025; Figure S2: (a–c) Brown rice rate, milled rice rate, and head rice rate; (d,e) RVA pasting profiles of rice starch under different treatments. Table S1: Analysis of variance for nitrogen accumulation in rice at different growth stages under slow-release fertilizer and straw returning; Table S2: Analysis of the effects of slow-release fertilizer and straw returning on the root morphology of rice during the tillering stage; Table S3: Analysis of variance for leaf SPAD values of rice at different growth stages under slow-release fertilizer and straw returning; Table S4: Effects of slow-release fertilizer and straw incorporation on net photosynthetic rate of rice leaves at different growth stages; Table S5: Effects of slow-release fertilizer and straw incorporation on stomatal conductance of rice leaves at different growth stages; Table S6: Effects of slow-release fertilizer and straw returning on intercellular CO_2_ concentration in leaves of rice at different growth stages; and Table S7: Effects of slow-release fertilizer and straw incorporation on transpiration rate of rice leaves at different growth stages.

## Abbreviations

The following abbreviations are used in this manuscript:

SSS	Soluble Starch Synthase
SBE	Starch Branching Enzyme
RVA	Rapid Visco Analyser
AGPase	ADP-glucose Pyrophosphorylase
GBSS	Granule-Bound Starch Synthase
Pn	Net Photosynthetic Rate
Gs	Stomatal Conductance
Tr	Transpiration Rate
Ci	Intercellular CO_2_ Concentration
N	Nitrogen
P	Phosphorus
K	Potassium
SPAD	Soil–Plant Analysis Development chlorophyll index
PKV	Peak Viscosity
HPV	Hot Paste Viscosity
BDV	Breakdown Viscosity
CPV	Cool paste Viscosity (final viscosity)
SBV	Setback Viscosity
PaT	Pasting Temperature
CSV	Consistency Viscosity
DAH	Days After Heading
DAF	Days After Flowering
S	Straw incorporation
F	Fertilizer treatment
