# Supplementary material for: Synergistic Enhancement of Rice Yield and Quality by Combined Slow-Release Fertilizer and Urea Under Straw Incorporation Through Optimized Grain Filling and Starch Biosynthesis
Source: Plants (Basel). 2026 Aug 25;15(17):2591. doi: 10.3390/plants15172591 (PMC13566953; doi:10.3390/plants15172591)
Supplement: Supplementary file 1 [file plants-15-02591-s001.zip › Tables S1-S7.pdf]

Table S1 Analysis of variance for nitrogen accumulation in rice at different growth stages under slow-release fertilizer and straw returning

Treatment	HS	FS	MS
S	ns	ns	**
F	**	*	**
S×F	**	ns	*

Table S2 Analysis of the effects of slow-release fertilizer and straw returning on the root morphology of rice during the tillering stage

Treatment	Length (m)	Average Diameter (mm)	Volume (cm^3)	Surface Area (m^2)
S	**	**	**	**
F	**	ns	ns	ns
S×F	ns	ns	ns	ns

Table S3 Analysis of variance for leaf SPAD values of rice at different growth stages under slow-release fertilizer and straw returning

Factor	TS	JS	HS	FS	MS
S	ns	ns	ns	ns	**
F	ns	**	**	*	**
S×F	*	**	*	ns	ns

Table S4 Effects of slow-release fertilizer and straw incorporation on net photosynthetic rate of rice leaves at different growth stages

Year	Treatment	TS (μmol m^{-2} s^{-1})	JS (μmol m^{-2} s^{-1})	HS (μmol m^{-2} s^{-1})	FS (μmol m^{-2} s^{-1})	MS (μmol m^{-2} s^{-1})
2024	N	17.95±0.07a	20.10±1.24a	19.30±0.85b	12.74±0.44c	4.10±0.74c
	N+S	17.18±0.03c	19.24±0.78a	21.43±1.43ab	15.20±0.65bc	7.74±1.15ab
	SRF	17.70±0.11b	19.46±1.48a	20.48±1.17ab	14.15±0.72bc	7.06±0.64b
	SRF+S	16.18±0.07e	17.13±1.79b	22.95±2.66a	15.71±1.61bc	8.47±0.81ab
	SRF:N(7:3)	17.96±0.28a	20.10±2.04a	20.94±1.23ab	16.30±1.21ab	7.80±0.22ab
	SRF:N(7:3)+S	16.60±0.12d	17.50±2.27b	21.51±0.73ab	19.29±0.81a	9.59±2.42a
2025	N	21.75±0.58ab	26.37±0.78a	24.16±0.52c	16.43±0.36c	4.44±0.43c
	N+S	21.73±0.65ab	24.90±0.97ab	26.64±0.88b	17.91±0.52abc	9.25±0.57ab
	SRF	20.34±1.53ab	22.88±0.37bc	25.69±1.10bc	16.75±042bc	8.77±0.19b
	SRF+S	19.15±0.61b	20.54±0.64c	27.07±0.40bc	18.34±0.88ab	9.86±0.76ab
	SRF:N(7:3)	23.51±1.56a	25.72±0.87a	27.79±0.34ab	17.04±0.26abc	9.19±0.19ab
	SRF:N(7:3)+S	21.09±0.67ab	22.20±0.56c	29.05±0.49a	18.56±0.52a	11.18±1.00a
	S	ns	**	*	ns	ns
	F	*	**	*	*	**
	S×F	ns	ns	*	ns	**

Table S5 Effects of slow-release fertilizer and straw incorporation on stomatal conductance of rice leaves at different growth stages

Year	Treatment	TS (mol m^{-2} s^{-1})	JS (mol m^{-2} s^{-1})	HS (mol m^{-2} s^{-1})	FS (mol m^{-2} s^{-1})	MS (mol m^{-2} s^{-1})
2024	N	0.45±0.03a	0.87±0.06a	0.56±0.01d	0.22±0.01c	0.07±0.01b
	N+S	0.39±0.01b	0.55±0.10b	0.71±0.07ab	0.25±0.05bc	0.10±0.04a
	SRF	0.44±0.03a	0.70±0.07ab	0.61±0.00cd	0.26±0.03bc	0.11±0.01a
	SRF+S	0.43±0.05ab	0.54±0.07b	0.78±0.01a	0.28±0.04bc	0.12±0.01a
	SRF:N(7:3)	0.44±0.02a	0.58±0.10b	0.63±0.04bcd	0.37±0.04a	0.08±0.01a
	SRF:N(7:3)+S	0.40±0.02ab	0.56±0.09b	0.66±0.09bc	0.39±0.07a	0.11±0.03a
2025	N	0.46±0.03a	0.68±0.09a	0.66±0.06b	0.56±0.09a	0.08±0.01b
	N+S	0.37±0.06ab	0.43±0.02b	0.75±0.07ab	0.63±0.09a	0.11±0.01ab
	SRF	0.36±0.03ab	0.67±0.07a	0.66±0.04b	0.63±0.10a	0.10±0.01ab
	SRF+S	0.29±0.03b	0.61±0.07ab	0.82±0.04ab	0.68±0.05a	0.12±0.01ab
	SRF:N(7:3)	0.44±0.03a	0.60±0.02ab	0.83±0.05ab	0.63±0.05a	0.11±0.02ab
	SRF:N(7:3)+S	0.37±0.03ab	0.56±0.09ab	0.93±0.07a	0.68±0.08a	0.15±0.07a
	S	ns	ns	ns	ns	ns
	F	*	ns	*	ns	ns
	S×F	ns	ns	ns	ns	ns

Table S6 Effects of slow-release fertilizer and straw returning on intercellular CO_2 concentration in leaves of rice at different growth stages.

Year	Treatment	TS (μmol mol^{-1})	JS (μmol mol^{-1})	HS (μmol mol^{-1})	FS (μmol mol^{-1})	MS (μmol mol^{-1})
2024	N	297.12±0.57c	296.81±8.62a	289.84±2.01a	290.71±3.01a	262.52±5.88a
	N+S	315.51±0.44a	311.46±3.49a	269.56±6.65b	273.33±20.22a	260.75±6.01a
	SRF	302.44±0.52bc	309.37±4.82a	284.94±4.59ab	259.42±18.64a	232.74±56.71a
	SRF+S	306.40±4.85b	320.12±3.33a	267.18±23.21b	247.27±12.80a	209.04±5.80a
	SRF:N(7:3)	303.11±3.55b	298.30±13.93a	277.82±5.57ab	280.11±17.22a	239.47±15.94a
	SRF:N(7:3)+S	305.79±4.80b	311.89±2.37a	272.48±2.37ab	271.71±19.76a	232.34±56.91a
2025	N	308.89±6.96a	286.71±3.14a	271.63±4.24a	295.41±2.84a	259.49±15.37a
	N+S	318.83±4.01a	295.26±0.86a	251.38±6.10ab	282.46±7.27a	255.42±48.71a
	SRF	314.39±3.63a	288.54±4.70a	264.34±3.92a	279.62±7.52a	240.86±24.95a
	SRF+S	315.20±5.11a	291.90±7.83a	234.24±13.48b	277.68±5.80a	222.59±16.60a
	SRF:N(7:3)	312.38±5.24a	289.02±3.28a	265.71±3.74a	287.23±13.29a	239.08±18.63a
	SRF:N(7:3)+S	319.76±3.84a	298.50±3.13a	240.84±6.76b	279.75±7.91a	195.68±37.40a
	S	ns	ns	ns	ns	ns
	F	ns	ns	ns	ns	ns
	S×F	ns	ns	ns	ns	ns

Table S7 Effects of slow-release fertilizer and straw incorporation on transpiration rate of rice leaves at different growth stages

Year	Treatment	TS (mmol m^{-2} s^{-1})	JS (mmol m^{-2} s^{-1})	HS (mmol m^{-2} s^{-1})	FS (mmol m^{-2} s^{-1})	MS (mmol m^{-2} s^{-1})
2024	N	6.20±0.22a	8.86±0.04a	8.27±0.98a	3.29±0.32c	1.85±0.20b
	N+S	5.51±0.37a	8.44±0.08a	9.480.92a	5.03±0.85bc	2.15±0.68a
	SRF	6.04±0.18a	8.65±0.48a	8.29±0.28a	4.82±0.58bc	2.28±0.10a
	SRF+S	5.84±0.25a	7.00±0.07b	10.29±0.19a	5.39±0.49bc	2.66±0.22a
	SRF:N(7:3)	6.15±0.63a	8.66±0.18a	8.91±0.27a	5.93±0.80a	1.94±0.34a
	SRF:N(7:3)+S	6.03±0.50a	7.19±0.20b	9.00±0.36a	7.10±0.39ab	2.50±0.66a
2025	N	8.15±0.65a	9.86±0.45a	8.46±1.57a	6.82±0.28c	1.45±0.13b
	N+S	6.26±0.17b	8.56±0.35a	9.15±1.31a	9.09±0.09a	2.43±1.05a
	SRF	6.90±0.24b	9.18±0.64a	9.03±1.76a	7.24±0.51c	2.20±0.14a
	SRF+S	6.47±0.19b	9.01±0.49a	9.95±0.33a	8.46±0.42ab	2.97±0.30a
	SRF:N(7:3)	7.19±0.16ab	9.45±0.16a	9.47±1.58a	7.54±0.16bc	2.10±0.20a
	SRF:N(7:3)+S	6.09±0.37b	9.36±0.30a	10.32±0.56a	7.67±0.22bc	2.43±0.37a
	S	ns	ns	ns	**	ns
	F	*	ns	ns	**	ns
	S×F	*	ns	ns	ns	ns
